# *Streptomyces zaomycetitus* strain GH90: a source of violet pigment with metabolic profiling and potential application in textile: in vitro supported by in silico studies and molecular docking

**DOI:** 10.1186/s12866-025-04329-1

**Published:** 2025-10-03

**Authors:** Gehad H. El Sayed, Mohamed Fadel, Mohamed Marzouk, Hend M. Ahmed, Nervana S. Diab, Ahmed A. Hamed

**Affiliations:** 1 Microbial Chemistry Department, Biotechnology Institute, National Research Centre, Dokki, Giza, Egypt; 2 Chemistry of Tanning Materials and Leather Technology Department, Chemical Industries Research Institute, National Research Centre, Dokki, Giza, Egypt; 3 Dyeing, Printing and Intermediate Auxilaries Department, Textile Research and Technology Institute, National Research Centre, Dokki, Giza, Egypt; 4 Children Hospital, Faculty of Medicine, Mansoura University, Mansoura, Egypt

**Keywords:** *Streptomyces* sp., Violet pigment, Fermentation conditions, Textile printing, And molecular docking

## Abstract

Dyes are used daily in the culinary, textile, paper, leather, and agricultural sectors. Synthetic colors pollute the environment and have harmful effects. Microorganism-produced pigments are more cost-effective and environmentally benign than animal, plant, and synthetic pigments. The present study focuses on isolating a pigment-producing actinomycete, a novel violet pigment produced by a local isolate strain identified as *S. zaomycetitus* GH90, using 16 S rRNA from Egyptian soil. Optimizing violet pigment yield necessitated the precise adjustment of various growth parameters. *Streptomyces zaomyceticus* GH90 achieved optimal production under specific conditions. pH 8.0, agitation at 180 rpm, temperature at 37 °C, 1.5% starch concentration, ammonium sulfate, dipotassium hydrogen phosphate, 2% inoculum, 20% medium volume, and a 10-day incubation period. Ethanol served as the solvent for pigment extraction. Thermal analysis indicated no degradation at 40 °C. At 50 °C, retention was 98.7% and remained stable at 97.5% when subjected to temperatures between 60 and 80 °C for 1 h. Retention remained above 91% at temperatures of 90 and 100 °C, indicating significant heat stability. The pigment preserved its color integrity in an aqueous solution at pH 8.0. The UV–Vis spectra exhibited a maximum wavelength (λmax) at 580 nm. LC–MS analysis revealed the presence of indole, scopoletin, myricetin, and phosphorylated compounds, which are likely contributors to color and bioactivity. The pigment exhibited superior textile dyeing characteristics, including vivid coloration, high durability, and significant antibacterial activity, establishing it as a versatile and effective dye for antimicrobial textiles. Moreover, the most prevalent molecule, has high permeability, moderate solubility, and selective enzyme interactions, making it a suitable therapeutic candidate. Molecular docking shows significant OMPA and OprD binding affinities, supported by hydrophobic, π-π stacking, and hydrogen bond interactions. The obtained results clearly show how important *Streptomyces* is as a source of bioactive metabolites with potential applications.

**Supplementary Information:**

The online version contains supplementary material available at 10.1186/s12866-025-04329-1.

## Introduction

Since pigments may restore lost color during production or enhance natural color, they play a role in several manufacturing operations. By providing a distinctive sensory element, pigments enhance customer appeal. Synthetic pigments can negatively impact human health, potentially harming critical organs including the brain, kidneys, liver, heart, and systems like the immunological, reproductive, or respiratory [1]. As stated by Sarmiento-Tovar et al. (2022), there are three fundamental sources of natural pigments: bacteria, plants, and animals [2]. The stability, year-round availability, and ease of cultivation of microbial pigments make them highly desirable [3, 4]. El Sayed et al. (2025) had red pigment from *Streptomyces* *phaeolivaceus *strain GH27 [3]. Sharma et al. (2025) had violet pigment from *Streptomyces* DP6 [4]. One of the most potential natural sources of food colorants is microorganisms, specifically bacteria, fungus, and microalgae. These organisms are capable of producing food colorants both extracellularly and intracellularly. Due to their ability to be cultivated all year round on food and agricultural wastes, they are distinguished by their high, sustainable productivity and affordable costs. Furthermore, the obtained pigments exhibit a wide range of properties, including easy extraction, relative heat stability, rapid growth in economic culture media, independence from environmental conditions, and a variety of colors and shades [5–8]. Due to the aforementioned, the manufacture of microbial pigments is currently one of the fastest-promising and developing research areas, with potential for a wide range of uses in industrial fields [9–11]. Pattapulavar et al. (2025) have isolated psi-carotene from the strain *Streptomyces sp* [10]. From an industrial perspective, however, it is necessary to develop a high-tech and economical method for the large-scale synthesis of diverse microbial pigments [12]. Despite the fact that there are numerous natural pigments, only a few are obtainable in large enough quantities for industrial use [12–14]. According to reports, *Streptomyces* is one of the most fascinating microbiological genera for pigment production [15–25]. Restaino et al. (2025) had melanin from *Streptomyces nashvillensis*. Sarmiento-Tovar (2024) has pigmented extracts from *Streptomyces* strains [20, 21].

Considering all the above-mentioned vital uses and industrial applications for safe microbial pigments in a variety of fields, alongside the more important activities, beneficial properties, and biotechnological potential of *Streptomyces*, the current study genetically identified a novel *S. zaomycetitus* strain GH90. The production conditions of its violet pigment were optimized, and the composition was physicochemically studied by UV and GC-MS spectrometry.

## Materials and methods

### Sample collection

Sample collection was carried out from different locations and fields of agriculture in Aswan, the Red Sea, Al Fayoum, Kafr El Sheikh, and other governorates [26]. After soil sample collection, the obtained samples were transferred into the laboratory in sealed, sterilized plastic bags, coded, photographed, and kept in a fridge at 4 °C until further processing.

### Isolation and screening of actinomycetes

Isolation of actinomycetes was carried out following the technique reported by El Sayed et al., 2023; actinomycete isolation happened by using starch nitrate agar medium [27]. Components of medium by (g/L): Starch (20), KNO_3_ (2.0), K_2_HPO_4_ (1.0), CaCO_3_, (3.0), MgSO_4_ (0.5), NaCl (0.5), FeSO_4_, (0.01), and pH 7.8 agar (20) were in the medium. Morphological and pigmentation screenings identified actinomycetes colonies on all plates. Streaking the actinomycete colony with substantial coloration onto new media yielded a pure culture.

### Cultural, chemical, and biochemical characterization of actinobacterial isolate GH90

The actinobacterial isolate GH90 was characterized using the International *Streptomyces* Project. GH90 was grown on a range of ISP media to examine cultural characteristics such as soluble pigments, sporulation, aerial and substrate mycelia color, etc. Numerous biochemical experiments were conducted, including the production of hydrogen sulfide, the reduction of nitrate, and the hydrolysis of casein, lipids, and starch. The trials also involved the use of ISP9 medium that included different types of carbohydrates. Finally, the diaminopimelic acid isomers in the cell wall and the sugar content of the whole-cell hydrolysate were investigated.

### Genomic identification of actinobacterial isolate GH90

The culture of bacteria underwent genomic extraction of DNA using the Gene JET Genomic DNA Purification Kit (Thermoscientific, USA) by the manufacturer’s guidelines. Genomic DNA was used to amplify the 16 S rDNA gene [28]. The 16 s rDNA gene fragment was amplified using the methodology of El-Sayed et al. (2018) with universal primers: forward primer sequence (5′AGAGTTTGATCCTGGCTCAG3′) and reverse primer sequence (5 CTACGGCTACCTTGTTACGA3-), and the PCR program was used: 95 °C for 3 min as the initial denaturation step, followed by 35 cycles of denaturation at 95 °C for 30 s, annealing at 55 °C for 1 min for 16 S rDNA gene amplification, and 60 °C for gene amplification, extension at 73 °C for 2 min, with a final extension at 72 °C for 10 min [29]. A multiple sequence alignment of 16 S rRNA sequences from this work and those obtained from GenBank was conducted using the MUSCLE method [30] MEGA X used [31]. The neighbor-joining approach deduced the evolutionary history [32].

### Preparation of chosen actinomycete inoculum

Actinomycetes slants with an age of five days were used for inoculation. A 1.5% spore suspension of actinomycetes slants with an age of five days was used to inoculate 250 ml conical flasks holding 50 ml of used media for the production of pigment. The used media consists of (g/L): starch, 20; KNO_3_, 2.0; K_2_HPO_4_, 1.0; MgSO_4_, 0.5; NaCl, 0.5; CaCO_3_, 3.0; FeSO_4_, 0.01; pH, 7.8. Subsequently, incubation occurred in a rotary shaker at 150 rpm for three days at 28 °C.

### Study of culture conditions for maximum production of pigment

The study examined the impact of various fermentation conditions, including *p*H (6, 6.5, 7, 7.5, 8, 8.5, and 9), shaking speed (50, 100, 150, 200 and 250), incubation temperature (25, 28, 31, 34, 37 °C, and 40 °C), different concentration of starch (carbon source) (0.5, 1.0, 1.5, 2.0, 2.5, 3.0, 3.5, 4.0, 4.5, 5.0%) (v/v), and nitrogen sources (Ammonium nitrate, Ammonium oxalate, Ammonium citrate, Potassium nitrate, Ammonium sulphate, Diammonium hydrogen sulfate, Diammonium hydrogen sulfate, Diammonium phosphate, Diammonium hydrogen phosphate, Diammonium citrate, Diammonium iron sulfate, Casein, Malt extract, Yeast extract, Yeast extract), phosphorous sources (K_2_HPO_4,_ KH_2_PO_4,_ Na_2_HPO_4,_ NaH_2_PO_4_, K_3_pO_4_), inoculum size (0.5-5%) (v/v), culture medium volume (25–125 ml) in a 250 ml conical flask, incubation period (3–12 days), and heavy metal concentrations. Inoculating *S. zaomycetitus* GH90 bacterial suspensions independently examined pigment synthesis.

### Extraction, and properties of the violet pigment

Upon completion of the fermentation phase, the whole biomass of the culture was filtered using Whatman No. 1 filter paper. The filtrate was then centrifuged at 10000 rpm for 10 min. Supernatants were deemed the origin of extracellular pigment. The pigment was extracted per the methodologies of Kazi et al. (2022). Where equal volumes of different solvents (ethanol, methanol, acetone, propanol, hexane, water, and supernatant sample) were taken and mixed well. The mixture was centrifuged at 8,000 rpm for 10 min; the supernatant was monitored at 540 nm to check the optical density. The extract was lyophilized and used for further characterization studies [33, 34]. The heat stability of the violet pigment was assessed using the methodology of Mok and Hettiarachchy. (1991) [35]. The impact of *p*H fluctuations on the stability of violet pigment was investigated as detailed by [36]. A UV-visible spectrophotometric experiment was conducted to ascertain the λmax range [3, 33].

### Liquid chromatography mass analysis of the pigment

Using a SCIEX Triple Quad 5500 + MS/MS system with electrospray ionization (ESI) for detection and an ExionLC AC system for separation, the sample was analyzed using liquid chromatography-electrospray ionization–tandem mass spectrometry (LC-ESI-MS/MS). An Ascentis^®^ Express 90 Å C18 Column (2.1 × 150 mm, 2.7 μm) was employed for the separation. Two eluents were used in the mobile phases: A: acetonitrile (LC grade); B: 5 mM ammonium formate, pH 3. A linear increase from 5 to 100% B from 1 to 20 min, 100% B from 20 to 25 min, 5% B at 25.01 min, and 5% B from 25.01 to 30 min comprised the configuration of the mobile phase gradient. The injection volume was 5 µl, and the flow rate was 0.3 ml/min. Using the following settings, a scan (EMS-IDA-EPI) with a range of 100 to 1000 Da for MS1 was performed in negative ionization mode for MS/MS analysis: Ion source gases 1 and 2: 45 psi; mass range for MS2: 50 to 1000 Da; delustering potential: 80 V; collision energy: 35 eV; curtain gas: 25 psi; ion spray voltage: 5500 V; source temperature: 500 °C.

### Gas chromatography/MS analysis of the pigment

A Trace GC-TSQ mass spectrometer (Thermo Scientific, Austin, TX, USA) equipped with a direct capillary column TG-5MS (30 m x 0.25 mm x 0.25 μm film thickness) was used to determine the chemical constituents of the samples. The temperature of the column oven was first maintained at 50 °C, then raised by 5 °C per minute to 250 °C, kept for 2 min, and then raised by 30 °C per minute to the ultimate temperature of 300 °C, held for 2 min. Helium was employed as a carrier gas at a steady flow rate of 1 ml/min, and the injector and MS transfer line temperatures were maintained at 270 and 260 °C, respectively. The autosampler AS1300 paired with GC in the split mode was used to automatically inject diluted samples of 1 µl after a 4-minute solvent delay [37].

### Printing of cotton, wool, and polyester fabrics with extracted pigment

This study examined various garments made from five different fabric types: cotton, wool, polyester, a 50/50 cotton/polyester blend, and polyamide. All fabrics except for polyamide.

The cotton garments from Cairo were subjected to a comprehensive scouring process using a specifically formulated aqueous solution with a liquor ratio of 1:50. This solution contained 2 g/L of high-quality hospital-grade nonionic detergent (Clariant) and was maintained at a temperature of 50 °C for a duration of 30 min. After thorough cleansing, the fabrics were rinsed with cold tap water and air-dried at room temperature. Wool: The wool fabrics underwent a mild yet efficient cleaning process using a solution of 2 g/L nonionic detergent (TERGITOL™ NP-9 Surfactant) at 50 °C for thirty min. Each piece underwent a thorough rinse followed by air drying in ambient conditions, thereby maintaining its inherent appeal. The polyester fabrics were quickly cleaned for 10 min in a special solution made of 3 g/L sodium carbonate, 0.5 g/L wetting agent, and 1 g/L synthetic detergent. The polyester fabrics were subjected to a swift scouring process of 10 min in a specialized solution containing 3 g/L sodium carbonate, 0.5 g/L wetting agent, and 1 g/L synthetic detergent. The bleached 50/50 cotton/polyester blend fabrics, sourced from EL-Nasr Company, were scoured using a calculated 1:50 liquor ratio with 2 g/L nonionic detergent (Clariant) and 1 g/L sodium carbonate. The process occurred at temperatures between 60 °C and 80 °C, which ensured optimal fabric integrity. Subsequently, the fabrics were rinsed with cold tap water and air-dried, revealing their inherent beauty. The study examined the properties of woven polyamide six fabrics (210 denier/35-strand yarns; NH₂—[(CH₂)₅—CONH] n—COOH; density: 1.14 g/cm³) obtained from El-Nasr Spinning, Weaving, and Knitting Company in Shourbagy, Cairo. The fabrics were treated with soap at 60 °C for 30 min, rinsed thoroughly, and air-dried at room temperature, preserving their texture for observation.

### Used chemicals

Nonionic detergent, urea, tannic acid, and ammonium persulfate (NH₄)₂S₂O₈ as thermal initiators are supplied from laboratory-grade chemicals. Thermal curing binders and thickeners were purchased from Sigma Aldrich.

The process of printing paste preparation.

1. We prepared a printing paste weighing 100 g, composed of the following components: 2 g of synthetic thickening agent, a binder between 5 and 20 g, 4 g of urea, 0.5 g of sodium dihydrogen phosphate, and 2 g of colorant, and water were adjusted in quantity to reach the desired viscosity.
2. Printing: Apply the prepared paste uniformly onto the fabric.
3. Fixing: Fix the print by thermal treatment at 180 °C for 3 min.
4. Washing procedure: Rinse thoroughly with cold water. Wash in hot water (60 °C) with 2 g/L of nonionic detergent. Rinse with hot water, and do the final rinse with cold water.
5. Drying: Allow the fabric to air-dry.
6. Color strength measurement: Evaluate and record the color strength of the printed fabric.

### Evaluation of color and fastness characteristics

In a thorough examination of the color dynamics of sensor wool textiles, we employed the advanced Ultra Scan PRO spectrophotometer, featuring a 10° standard viewer and D65 illuminant (Hunter Lab, USA). This sophisticated analysis not only assessed tinctorial strength (K/S) but also delved into CIE Lab metrics, which offer a vivid portrayal of color perception. The L* value captures the transition from deep blackness (0) to pristine whiteness (100), while the a* value gracefully navigates the spectrum from cool greenness (-) to warm redness (+). Meanwhile, the b* value reveals the delicate balance between blueness (-) and yellowness (+), enriching our understanding of the textile’s chromatic characteristics.

To ensure a comprehensive understanding of colorfastness, we meticulously followed acclaimed AATCC Test Methods: Test Method 8-2016 for rubbing [38], Test Method 61-2013 for washing [39], Test Method 15-2013 for sweat [40], and Test Method 16.1–2014 for light [41].

### Evaluation of UV protection

The ultraviolet protection factor (UPF) of both treated and untreated samples was meticulously evaluated in accordance with the esteemed Australian/New Zealand standard (AS/NZS 4366 − 1996). This evaluation classifies UPF into distinct categories: “good” (UPF 15–24), “excellent” (UPF 25–39), and “exceptional protection” (UPF greater than 40) against the harmful effects of UV radiation. This classification not only highlights the effectiveness of the treatments but also underscores the importance of safeguarding against UV exposure.

### Determination of antimicrobial activity of treated fibers

A set of test microorganisms was used for antimicrobial screening [3]. Overall, 180 µL of culture medium (bacterial lysogeny broth) was mixed with 10 µL of compounds (250 ug/ml). At the log phase, 10 µL of bacterial or fungal suspension was added. A Spectrostar Nano Microplate Reader (BMG Labtech GmbH, Allmendgrun, Germany) was used to measure the absorbance at OD 600 following an overnight incubation of the plates at 37 °C. Following the preparation of eight serial dilutions (250, 125, 62.5, 31.25, 15.63, 7.81, 3.90, and 1.95 µg/mL), further dilutions buffering the lowest concentration with no discernible growth of bacteria or fungus were tried in increments of 1 µg/mL based on the findings obtained. MICs are the average of the lowest concentrations at which, according to three different studies, no discernible bacterial or fungal growth took place. The positive control was ampicillin.

### ADME prediction

The ADME predictions for the TiO_2_ molecule were conducted using the methods described by Swanson et al. (2024) [42].

### Docking study

A molecular docking study was conducted to examine the binding interactions between indole and the *E. coli* outer membrane protein A (OMPA) (PDB ID: 1BXW), as well as the enhanced crystal structure of Pseudomonas aeruginosa OprD (PDB ID: 3SY7). The crystal structure of OMPA was sourced from the Protein Data Bank, and preparation involved the removal of water molecules and heteroatoms utilizing AutoDockTools software. Indole was generated with ChemDraw, and its three-dimensional structure was optimized utilizing the MM2 force field. The ligand and receptors were subjected to energy minimization to ensure stability. The docking procedure utilized AutoDock Vina, with the grid box configured to encompass the binding site of OMPA and OprD, informed by established active residues and the ligand’s location. The docking grid was set up to allow for ligand flexibility, whereas the protein was maintained as a rigid entity. The docking parameters, such as exhaustiveness and the number of docking poses, were optimized to maximize the accuracy of binding predictions. The interactions between indole, OMPA, and OprD were examined, emphasizing hydrophobic contacts, hydrogen bonds, and π-π stacking interactions. The binding affinity (in kcal/mol) was calculated, and results were visualized with PyMOL to identify key residues involved in binding.

### Statistical analysis

Statistical analysis was performed by calculating the standard deviation (SD) of triplicate measurements to ensure the reliability and reproducibility of the experimental data.

## Results and discussion

### Sample collection

According to El Sayed et al. (2025), 37 soil samples were taken using a sterile spatula from Egypt’s agricultural land at a depth of 0–10 cm and kept in high-density polyethylene bags that have been sterilized [3]. 

### Isolation and screening of actionobacterial colonies for ability to produce pigment

**Fig. 1:**
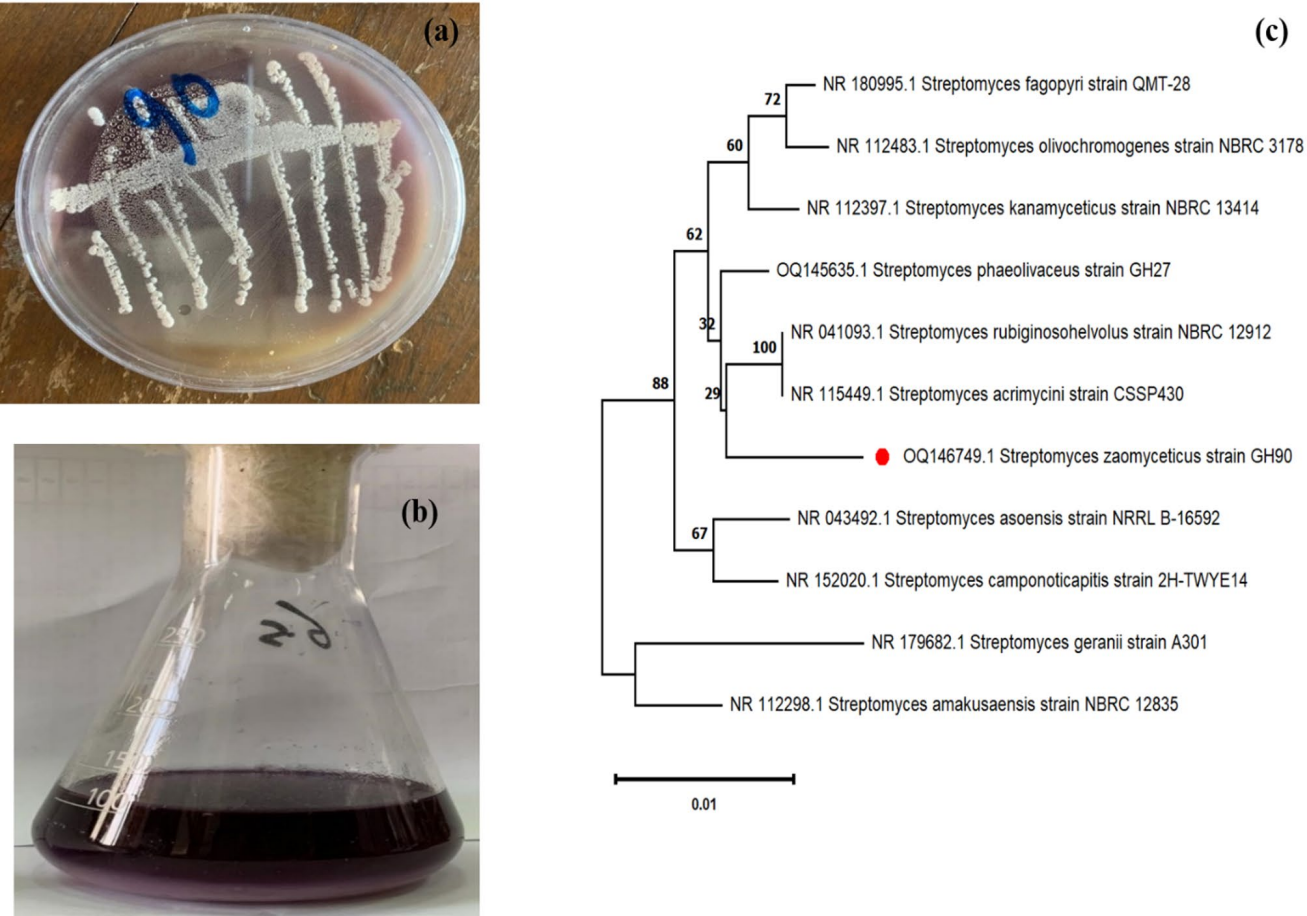
**a** Isolated colony morphology. **b** Violet Pigment produced by *S. zaomycetitus* GH90. **c** Phylogenetic tree of *S. zaomycetitus* GH90 based on 16 S rRNA gene sequences

Gram-positive filamentous bacteria, or prokaryotic microorganisms, are known as actinobacterial isolates. The rhizosphere of plants in farmed areas, sacred and marine waters, and other natural ecosystems are examples of its natural habitats. On the basal starch nitrate medium, 145 actinobacterial colonies were selected at random and isolated from various soil sources in Egypt (Table 1). The *S. zaomycetus* GH90 strain was chosen for additional research because it was a potent source of violet pigment among the 145 actinobacterial isolates. (Fig. 1a, b).

**Table 1:** Morphological characteristics of actionobacterial isolates having pigmentation ability

Isolate number	Spore mass	Substrate mycelium	Diffusible pigment
GH24	brown	Black	Black
GH43	Yellow	Faint brown	Faint brown
GH57	Grey	Colorless	Yellow
GH90	Yellow	colorless	Dark violet
GH104	Grey	Dark brown	Brown

On a variety of ISP media, cultural features of isolate GH90 were examined. GH90 grew on media from ISP1, ISP2, ISP3, ISP4, and ISP5. On the other hand, neither Tyrosine Agar (ISP-7) nor Peptone Iron Agar (ISP-6) medium showed any synthesis of melanin pigment (Table 1). GH90 can utilize mannitol, lactose, galactose, and starch as carbon sources. The presence of LL-diaminopimelic acid as the distinctive diamino acid in the cell wall was discovered by chemotaxonomic research. There was no distinctive sugar found in the whole-cell hydrolysate. Along with producing a variety of extracellular enzymes, such as lipase, amylase, protease, and xanthinase, GH90 has also shown positive results for nitrate reduction and H₂S production.

Strain GH90 was assigned to the *Streptomyces* genus based on morphological, cultural, and chemotaxonomic analyses. 16 S rRNA sequencing provided additional support for this categorization. Under accession number OQ146749.1, the *Streptomyces* isolate found in this research project was added to the NCBI Gene Bank database. The PCR amplification sequence that was obtained was 100% identical to *S. zaomycetus* GH90 (Fig. 1c). There is 99–100% similarity between the GH90 sequence and other *Streptomyces* species. The isolated strain GH90 was classified by 16 S rRNA gene sequencing phylogenetics. Figure 1c shows that the neighbor-joining tree groups the isolate in *Streptomyces* GH90 formed a separate clade with good bootstrap support due to its maximum sequence similarity to *Streptomyces zaomyceticus* (accession number OQ146749.1). This tight relationship identifies *Streptomyces zaomyceticus* GH90 as the pigment-producing isolate. It also differs from near-related species like *S. rubiginoshelvolus*,* S. acrimycini*, and *S. phaeolivaceus*, which were classified in surrounding branches. The evolutionary link emphasizes the isolate’s genetic distinctiveness and aids bioactive chemical synthesis research.

### Study of fermentation parameters for maximum production of pigment

**Fig. 2:**
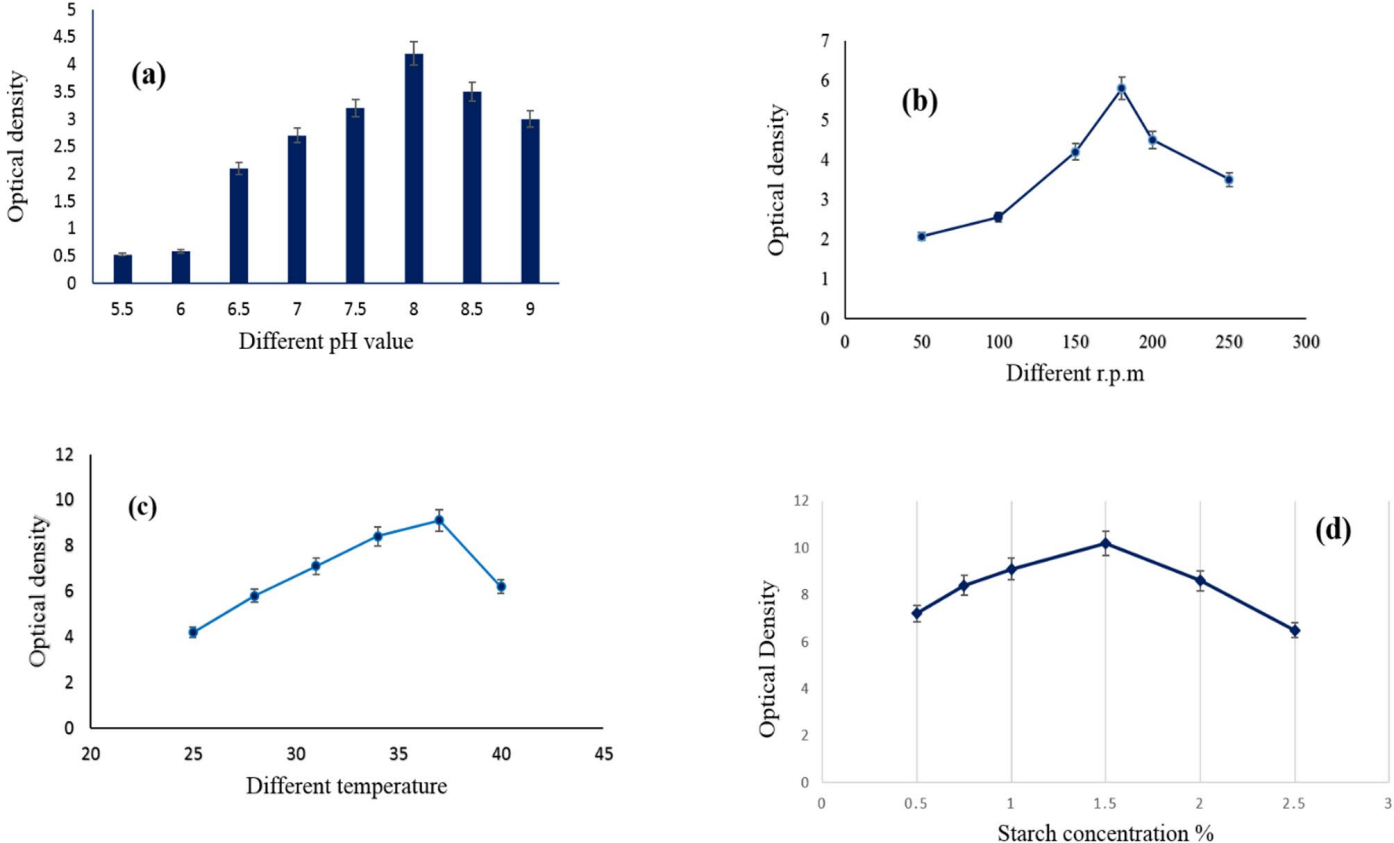
**a** Effect of different pH value, **b** Effect of incubation temperature, **c** Effect of (r.p.m) and **d** Effect of different starch concentration on the violet pigment production by *S.zaomycetitus* GH90. Rotation per minute (r.p.m)

The findings in Fig. 2a show that the newly discovered isolated strain of *S. zaomycetus* GH90 had its greatest pigment concentration at pH 8.0 and its maximum pigment density at a pH range of 7.5–9.0. Below 7.0, the concentration synthesis of violet pigment is adversely impacted. When investigating a novel isolate of actinomycetes for a particular pigment synthesis, it’s critical to examine the initial pH value. The hue of the pigment that the same organisms produce may be changing due to the growing medium’s pH. However, certain bacteria may produce significant quantities of red, yellow, and violet pigments, especially those belonging to the genus *Paecilomyces* [43]. The findings indicated that optimal development and pigment output were achieved at a shaking speed of 180 rpm (Fig. 2b). This outcome may be attributed to aeration, which is crucial for cellular development efficiency by enhancing the transfer of substrates and oxygen [44]. Ali et al. 2011 also observed the same results, optimal pigment synthesis occurred at 180 rpm [45]. Conversely, yeast (*Rhodorula* sp. and *Phaffia* sp.) exhibited optimal growth and pigment production in an agitation range of 180–900 rpm [46]. Temperature is one of the most important environmental factors affecting the growth of microorganisms; besides, it causes changes in many biosynthetic pathways. The study of the temperature of new isolates added to *p*H must come firstly at the beginning of studies. Figure 2c illustrated that the maximum violet pigment production was recorded by organism incubation at a temperature of 37 °C, while a negative effect was observed above that temperature. Many authors determined various temperatures for the highest pigment production from different actinomycetes strains. *Salinicoccus sp.* M. KJ997975 showed maximum pigment productivity at a temperature of 30 °C [3]. At the optimal temperature, enzyme activity seems to be augmented for pigment synthesis; hence, the peak pigment production was recorded at this temperature. Figure 2d demonstrated that the production of violet pigment in the growth medium infected with *S. zaomycetitus* GH90 increased with the addition of starch, rising from 0.5 to 1.5% (w/v), but thereafter declined with further starch increments. Starch is extensively used as a carbon source for the separation and pigment synthesis by actinomycetes [47]. Different actinomycetes produce pigment when grown in media containing various carbon sources [48–50].

**Fig. 3:**
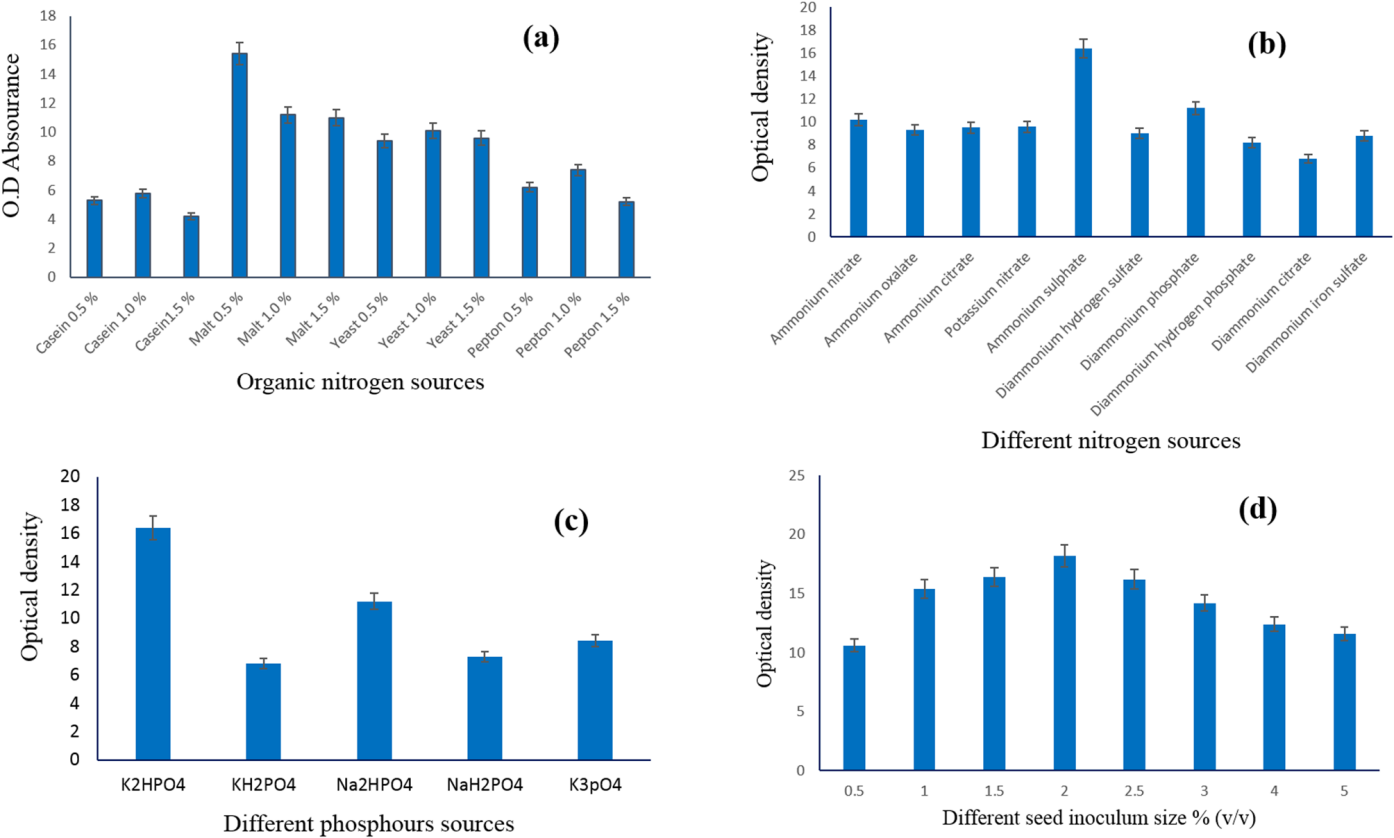
**a** Effect of organic nitrogen sources, **b** Effect of nitrogen sources, **c** Effect of different phosphorus sources, and **d** Effect of different seed inoculum size % (v/v) on the violet pigment production by *S. zaomycetitus* GH90

Out of the four sources examined, Fig. 3a shows that the *S. zaomycetus* GH90 strain produced violet pigment more effectively when there was 0.5% malt extract in the medium. This outcome was comparable to what [51], found. Numerous researchers discovered that the culture medium’s organic sources created more colors [51]. Figure 3b revealed that ammonium sulfate was more suitable than other tested organic sources for violet pigment production by the *S. zaomycetitus* GH90 strain. Many researchers reported different nitrogen sources for the production of pigments by various strains of actinomycetes [47–50, 52, 53]. Figure 3c shows that the production of violet pigment by the *S. zaomycetitus* GH90 strain is affected by the source of phosphorous involved in the culture medium. The data revealed that the di-potassium hydrogen phosphate was the more suitable source of phosphorus, i.e., supplement in growth medium to produce the maximum pigment density by *S. zaomycetitus* GH90 strain in the growth medium. However, potassium dihydrogen phosphate resulted in low pigment production, which can be discussed in light of the phosphorus source, affecting the growth medium’s pH. Many researchers applied different phosphorous sources in the culture media of actiomycetes [54]. The inoculum size percent is an important factor for accelerating carbon and other nutrient utilization in the culture medium. Figure 3d illustrates that inoculating the medium with 2% (v/v) inoculums gave the maximum violet pigment production than the other sizes of inoculums tested. The study of inoculum percent was taken into consideration when pigment was produced from microorganisms [55, 56]. High inoculum sizes led to an increase in biomass and decreasing pigment production due to increased biomass inhibiting the affinity of utilization of essential nutrients in the culture medium [57].

**Fig. 4:**
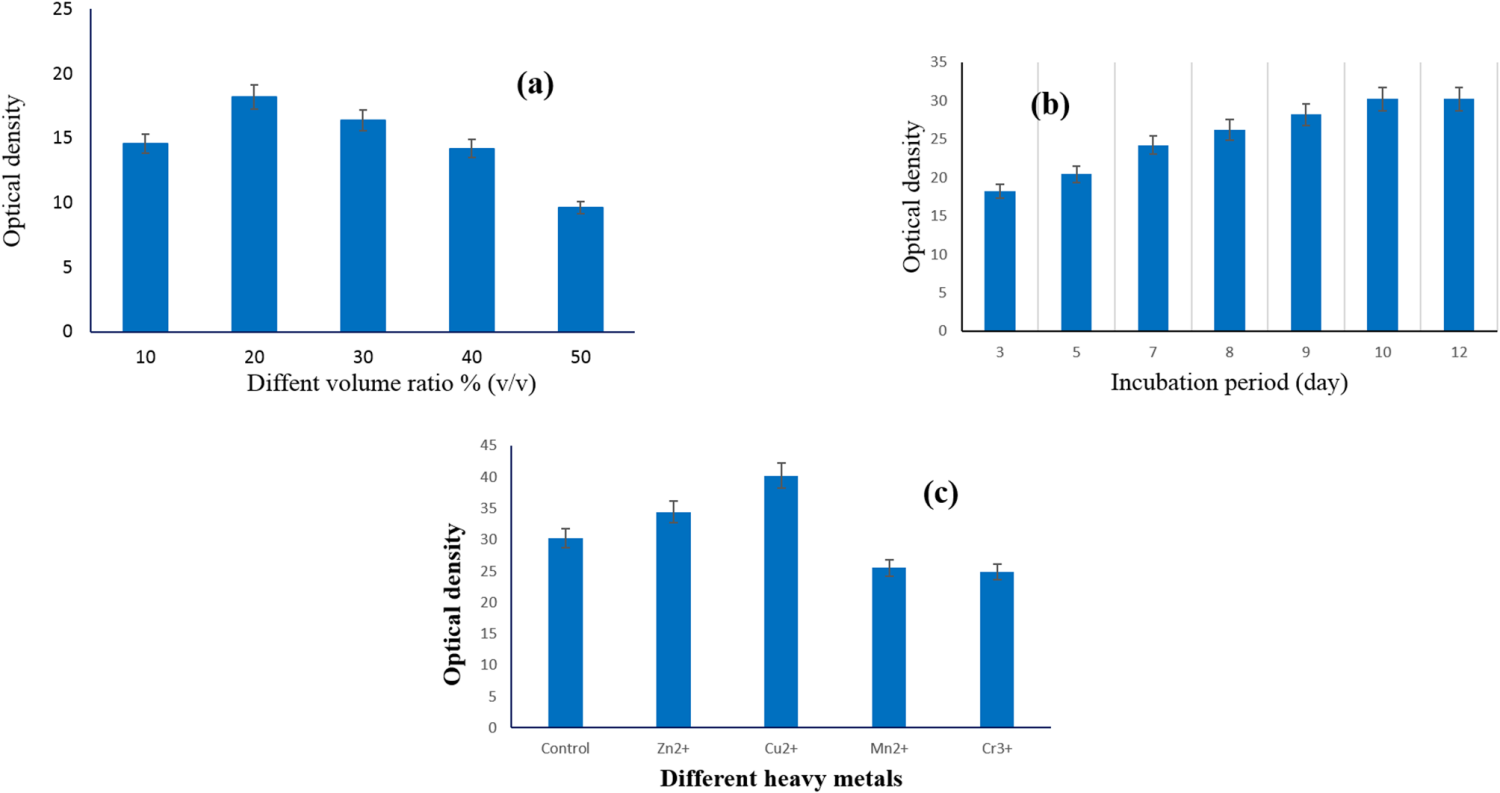
**a** Effect of volume ratio % (v/v), **b** Effect of incubation period (day), and **c** Effect of different heavy metals on the violet pigment production by *S. zaomycetitus* GH90

Different volumes of pigment production medium, in the range of 10–50% (v/v), were introduced in 250-ml conical flasks and sterilized by autoclave. Figure 4a exhibits that 20% (v/v) was the best volume for violet pigment production by *S. zaomycetitus* GH90. The density of violet pigment, released by *S. zaomycetitus* GH90 in the growth medium, increased by increasing the fermentation period up to ten days and then became constant (Fig. 4b). Elattaapy and Selim (2020) had maximum pigment production after 8 days; then, the production significantly decreased by increasing the incubation period [58]. Silbir and Goksungur (2019) obtained maximum pigment production by *Monascus purpureus* after 7 days of incubation [59].

Previous studies reveal that the optimum incubation time for maximum pigment production varies from one strain to another. Figure 4c showed that Cu ^+ +^ ion raised the violet pigment production by *S. zaomycetitus* GH90, considered an essential component in the culture medium, followed by Zn^++^. While El Sayed et al. (2023) had Cr ^+++^ ion as an important component for green pigment production by *S. nigra* GH12 [51].

### Extraction and properties of pigment

**Fig. 5:**
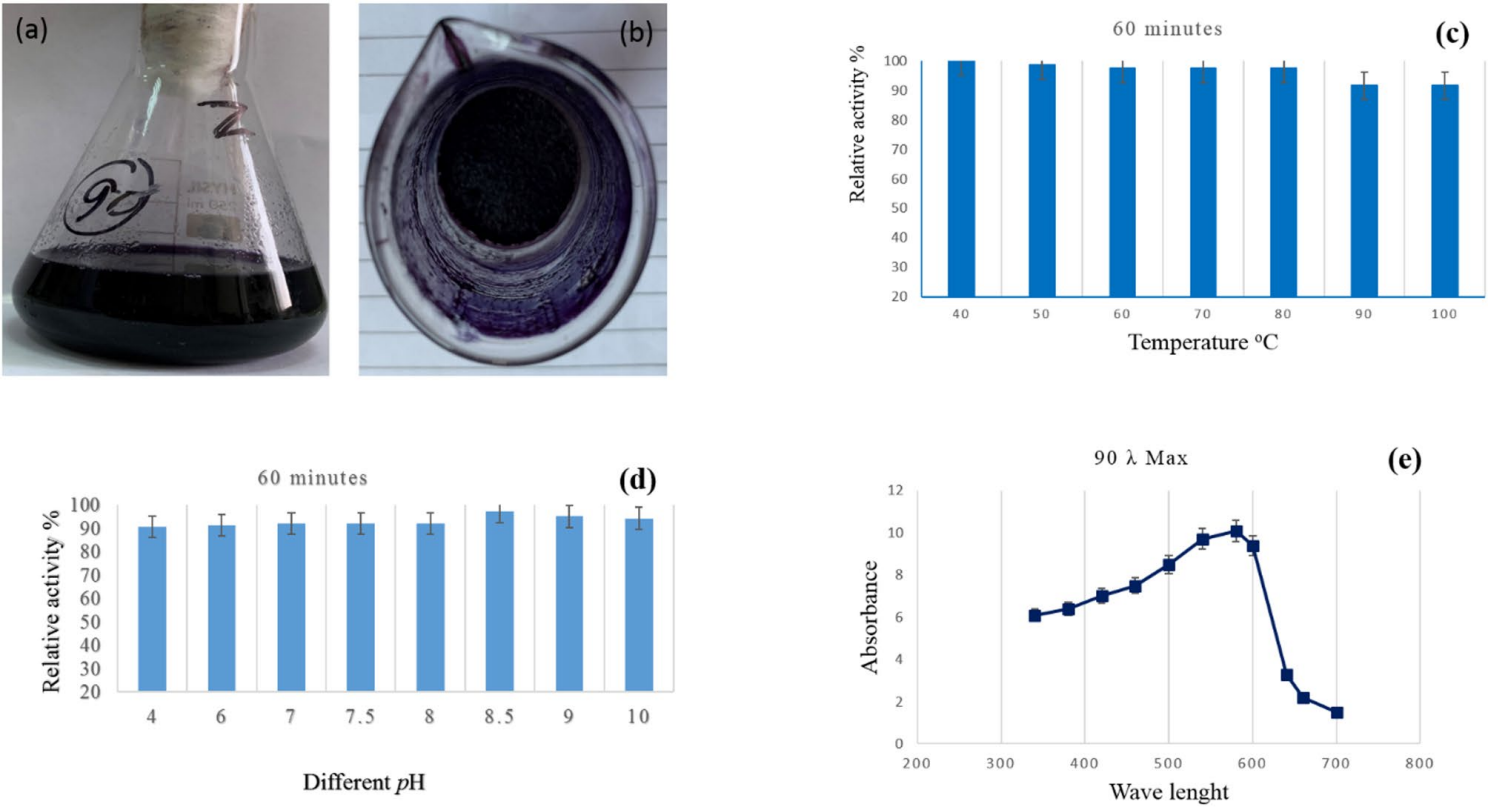
**a** Violet pigment extracted by ethanol solvent, **b** dried pigment after ethanol extraction, **c** heat treatment effect on violet pigment stability, **d** effect of pH on retention of brown pigment, **e** the UV absorption spectrum of extracted violet pigment

In actuality, the liquid-liquid extraction technique employing ethanol as an extract solvent yields the highest-yield extract of violet pigment. As a result, a lower pressure was used to vacuum dry the recovered extract. (Fig. 5a, b; Table 2). Heat treatment has a significant impact on the violet pigment’s stability. The pigment concentration did not significantly decrease at 40 °C. Up to 98.7% of the pigment was retained when the heating temperature was raised to 50 °C. The pigment retention was fixed at 97.5% when the heating temperature was raised to 60, 70, and 80 °C. By raising the temperature to 90 and 100 °C, the retention rises to 91.7 (Fig. 5c). In aqueous conditions, the violet pigment remained most stable at pH 8.0 (Fig. 5d). The green pigment generated by *S. nigra *had the maximum stability at pH 8.5 [51]. Figure 5e displays the violet pigment extract produced by *S. zaomycetus* GH90’s UV absorption spectrum. The data showed that the violet pigment extract’s absorption maximum peak had a λmax at 580 nm. Roselle pigments have been shown to have a λmax of 520 [60]. But according to El Sayed et al. (2023), the green pigment that *S. nigra* produces has a λmax of 340 nm [51].

**Table 2:** Violet pigment extraction produced by *S. zaomycetitus* GH90 isolate using different solvents

Extracted solvent	Pigment yield (µg/50 ml)
Ethanol	362
Acetone	218
Methanol	321

### LC-mass characterization of violet pigment produced by S. zaomycetitus GH90

In the current study, the LC-MS analysis of negative and positive modes data from the *S. zaomyceticus* strain GH90 identified numerous prominent metabolites, with a select number emerging as probable contributors to pigment and bioactivity (Supplementary Tables 1 and Table 2). One of the most notable discoveries was indole, identified at a retention time of 2.31 min, with a peak area of 1.14 × 10⁹, indicating its prevalence. Indole and its derivatives serve as precursors to several bioactive chemicals and may influence the resultant pigmentation or biological activity. Orotic acid (RT 2.14 min, area 1.24 × 10⁸) and its sodium adduct (RT 2.14 min, area 1.40 × 10⁸) were identified, both serving as intermediates in nucleotide biosynthesis and potential precursors for more complicated molecules. Scopoletin, identified at retention times of 4.62 min and 9.62 min, with peak areas of 1.19 × 10⁸ and 2.41 × 10⁸, respectively, is a hydroxycoumarin derivative that may enhance the strain’s antioxidant or antibacterial characteristics. Quisqualate, identified at retention times of 2.39 min (area 4.90 × 10⁸) and 10.18 min (area 9.16 × 10⁷), is a recognised excitatory amino acid that may contribute to the production of more intricate colours or bioactive compounds. Other significant metabolites comprised uracil (RT 2.76 min, area 1.59 × 10⁸), adenine (RT 2.39 min and 18.22 min, area 4.78 × 10⁷ and 4.55 × 10⁷), and nicotinic acid (RT 2.57 min, area 3.49 × 10⁸), all of which are integral to cellular metabolic pathways and may contribute to sondary metabolite synthesis. Myricetin (RT 2.96 min, area 1.20 × 10⁸) emerged as one of the most intriguing metabolites, recognised for its pigmentation and antioxidative properties. This may be a contender for the violet pigment present in this strain. Likewise, zearalenone (RT 2.96 min, area 1.03 × 10⁸), a molecule generated from polyketides, may affect pigmentation or biological activity according to its structural characteristics. Capsaicin, identified at a retention time of 15.73 min with an area of 7.38 × 10⁷, is often linked to pungency but may also influence pigmentation or antibacterial properties of the strain. The research identified many phosphorylated metabolites, including inosine-5′-monophosphate (RT 9.71 min, area 7.29 × 10⁷) and uridine-5′-diphospho-N-acetylgalactosamine (RT 9.99 min, area 1.03 × 10⁸), which may function as intermediates or regulatory entities in color. Several studies have used LC-MS for pigment characterization. Salim et al. [50]. For fungal pigment analysis, liquid chromatography with high-resolution electrospray ionization mass spectrometry (LC/HRESI-MS) was used as a reliable analytical method. Used LC/HRESI-MS to evaluate *Talaromyces satroroseus* TRP-NRC′s red pigment mixture and found several azaphilone derivatives with different m/z ratios. This method proved the pigment’s structural complexity and mass determination accuracy, enabling its use in biotechnological procedures, including textile dyeing and dermatological safety assessments. Such analytical methods are reliable for assessing pigment content and purity in comparable fungal metabolites.


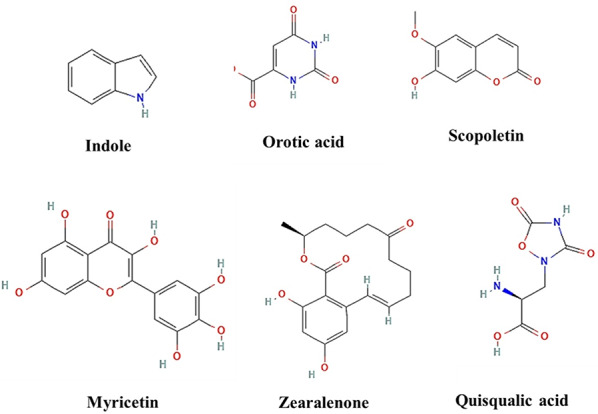


### GC/MS characterization of violet pigment produced by GH90

**Fig. 6:**
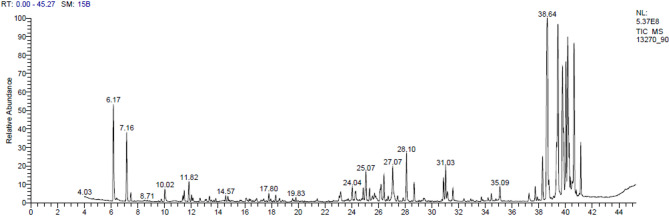
TIC chromatogram for total violet pigment extract of *S. zaomycetitus* GH90 isolate

During last decade, huge research efforts have been given for extensive chemical and biological evaluation for a wide-range of microbial pigments because of their biosafety and broad pharmaceutical, textile, and food applications [2, 61, 62]. Moreover, many research groups have paid specific attention to optimize the production conditions of such intrinsic pigments [34, 59, 63] and their biological investigation as antioxidant, antitumor, antimicrobial, and antiviral active agents [34, 64–70]. Recently the characterization of various microbial bioactive total pigment extracts and/or isolated pure metabolites produced by a large number of *Streptomyces* sp. have been performed with UV-, IR-, Ramman- and/or NMR analyses (Kaaniche et al., 2020 [2, 34, 65–69, 71, 72]. Furthermore, the hyphenation of chromatographic interfaces, i.e. GC-, HPLC with the advanced HRMS spectrometers of the soft ionization techniques (e.g. ESI, TSP, APCI), became the main and most rapid and accurate chemical characterization tool of the natural extracts, and microbial bio-pigments by the aid of the enrolled and established library database software programs [50, 51, 64, 68, 70, 73]. Chen et al., 2018 used both GC/and HPLC/MS spectrometry for identification of a large number of compounds from potent antitumor active extract of *S. nigra sp.* [71]. In the current study GC/MS analysis identified 41 compounds in the form of their silylated derivatives (Table 3) from the target violet pigment of the investigated new *S. zaomycetitus* GH90 on the basis of matching the R_*t*_-values, fragmentation pattern, corresponding MS-spectra, and other output parameters with those of WILEY 09 and NIST 14 mass spectral library database [37]. The identified metabolites were found to belong to mono-, di-saccharides, amino- and fatty acids. Figure 6 displayed the total ion chromatogram (TIC) that showed how crowded the target pigment extract was with different types of natural organic metabolites. It is worthy that 18 mass peaks were recorded for major metabolites (Figure. 6), including 8 of relative concentrations higher than 3% (1, 2, 34–37, 39, 40) together with 10 silyl derivatives observed for other metabolites occurred in a relative concentration more than 1% (7, 15, 21, 23, 24, 26, 27, 31, 38 and 41). The most major three identified metabolites were of disaccharide type-structures, i.e. Maltose **34**, Mannobiose **35** and Lactose **36** with R_*t*_-values bigger than 38.0 min along with Lactic acid (**1**, R_*t*_= 6.17 min, 4.7%) and L-Alanine (**2**, R_*t*_= 7.16 min, 3.41%), representing the major hydroxy organic and amino-acids.

**Table 3:** Identified silylated derivatives of the constitutive metabolites in the Violet pigment of *S. zaomycetitus* GH90 isolate

*P*.No.	*R*_t_, min	RC, %	MF	MW	Chemical name
1	6.17	4.70	C_9_H_22_O_3_Si_2_	234	Bis(trimethylsilyl)-lactic acid
2	7.16	3.41	C_9_H_23_NO_2_Si_2_	233	L-Alanine, *N*-(trimethylsilyl)-, trimethylsilyl ester
3	7.47	0.41	C_8_H_21_NO_2_Si_2_	219	Glycine, *N*-(trimethylsilyl)-, trimethylsilyl ester
4	10.02	0.65	C11H_27_NO_2_Si_2_	261	L-Valine, *N*-(trimethylsilyl)-, trimethylsilyl ester
5	11.37	0.25	C_9_H_27_O_4_PSi_3_	314	Silanol, trimethylsilylphosphate
6	11.47	0.73	C_12_H_29_NO_2_Si_2_	275	L-Leucine, *N*-(trimethylsilyl)-, trimethylsilyl ester
7	11.82	1.08	C_14_H_29_NO	308	Myristamide
8	12.02	0.30	C_12_H_29_NO_2_Si_2_	275	L-Isoleucine, *N*-(trimethylsilyl)-, trimethylsilyl ester
9	14.57	0.24	C_13_H_33_NO_3_Si_3_	335	L-Threonine, *N*,* O*-bis(trimethylsilyl)-, trimethylsilyl ester
10	14.74	0.25	C_11_H_28_O_3_Si_3_	292	Tris(trimethylsiloxy)ethylene
11	17.80	0.38	C_14_H_33_NO_3_Si_3_	347	*(E)-*L-Hydroxyproline, 1-(trimethylsilyl)−4-[(trimethylsilyl)oxy]-trimethylsilyl ester
12	18.32	0.32	C_14_H_32_O_5_Si_3_	364	Pentanedioic acid, 2-[(trimethylsilyl)oxy]-bis-(trimethylsilyl) ester
13	23.07	0.63	C_14_H_31_BO_6_Si_2_	362	α-D-Galactopyranoside, methyl 2,6-bis-O-(trimethylsilyl)-, cyclicmethylboronate
14	23.17	0.59	C_18_H_36_N_2_O_6_Si_3_	460	2′,3′,5′-Tris-O-(trimethylsilyl)-uridine
15	24.04	2.87	C_19_H_46_O_6_Si_4_	482	Methyl 2,3,5,6-tetrakis-O-(trimethylsilyl)-α-D-glucofuranose
16	24.86	0.57	C_21_H_52_O_6_Si_5_	540	1,2,3,4,6-Pentakis-O-(trimethylsilyl)-D-glucopyranose
17	25.34	0.57	C_21_H_52_O_6_Si_5_	540	1,2,3,4,6-Pentakis-O-(trimethylsilyl)-L-altrose
18	25.70	0.35	C_18_H_31_NO_3_Si_3_	393	Trimethylsilyl1-(trimethylsilyl)−5-[(trimethylsilyl)oxy]−1 H-indole-2-carboxylate
19	26.13	0.45	C_21_H_52_O_6_Si_5_	540	Pentakis(trimethylsilyl)α-D-allopyranose
20	26.19	0.66	C_21_H_52_O_6_Si_5_	540	1,2,3,4,6-Pentakis-O-(trimethylsilyl)-α-D-Talopyranose
21	26.42	1.28	C_22_H_55_NO_6_Si_5_	569	(1*Z*)-O-Methyloxime-2,3,4,5,6-Pentakis-O-(trimethylsilyl)-D-glucopyranose
22	26.73	0.23	C_22_H_55_NO_6_Si_5_	569	(1*E*)-O-Methyloxime-2,3,4,5,6-Pentakis-O-(trimethylsilyl)-D-allose
23	27.07	2.03	C_13_H_32_O_4_Si_3_	378	Tris-(trimethyl silyl)-levoglucosan
24	27.44	2.61	C_19_H_40_O_2_Si	328	Trimethylsilyl-palmitate ester
25	28.67	0.89	C_19_H_46_O_6_Si_4_	482	Tetrakis-O-(trimethylsilyl)-α-D-methylgalactose
26	30.87	1.05	C_21_H_40_O_2_Si	352	Trimethylsilyl-(9*Z*,12*Z*)-octadecadienoic acid ester
27	31.03	1.54	C_21_H_42_O_2_Si	354	Trimethylsilyl-(11*E)*-octadecenoic acid ester
28	31.16	0.51	C_21_H_42_O_2_Si	354	Trimethylsilyl-(9*E)*-octadecenoic acid ester
29	31.57	0.61	C_21_H_44_O_2_Si	356	Trimethylsilylstearic acid ester
30	34.45	0.35	C_36_H_86_O_11_Si_8_	918	Lactose, octakis(trimethylsilyl)ether, isomer 1
31	35.09	1.11	C_36_H_86_O_11_Si_8_	918	3-α-Mannobiose, octakis(trimethylsilyl)ether, isomer 1
32	37.26	0.43	C_26_H_17_N_3_	371	Pyridazinyl-1,2-bibenzoylbenzo[E]indolizine
33	37.73	0.68	C_17_H_42_O_5_Si_4_	438	D-Ribofuranose, tetrakis-O-(trimethylsilyl) ether
34	38.28	14.00	C_36_H_86_O_11_Si_8_	918	Maltose, octakis(trimethylsilyl)ether, isomer 1
35	38.64	14.01	C_36_H_86_O_11_Si_8_	918	2-α-Mannobiose, octakis(trimethylsilyl)ether
36	38.76	13.38	C_36_H_86_O_11_Si_8_	918	α-D-Lactose, octakis(trimethylsilyl)ether, isomer 2
37	39.77	7.35	C_36_H_86_O_11_Si_8_	918	Maltose, octakis(trimethylsilyl)ether, isomer 2
38	39.86	2.45	C_36_H_86_O_11_Si_8_	918	D-(+)-Cellobiose, octakis(trimethylsilyl)ether
39	40.03	6.14	C_36_H_86_O_11_Si_8_	918	D-(+)-Turanose, octakis(trimethylsilyl)ether
40	40.63	8.56	C_36_H_86_O_11_Si_8_	918	3-α-Mannobiose, octakis(trimethylsilyl)ether, isomer 2
41	41.14	2.29	C_36_H_86_O_11_Si_8_	918	2-α-Mannobiose, octakis(trimethylsilyl)ether, isomer 1

R_t_ (retention time); Rc (relative concentrations), MF (Molecular formula), MW (Molecular weight)

### Printing of cotton, wool, and polyester fabrics with extracted pigment

#### Color strength

This study presents compelling insights into the color strength of textiles printed with a vibrant violet pigment sourced from the S. *zaomycetitus* strain GH90. We focus on essential parameters such as the color coordinates (L*, a*, b*), ΔE (color difference), and K/S value (color strength), all vital for assessing the pigment’s effectiveness across various fabric types, including cotton, wool, polyester, polyamide, and cotton/polyester blends. Table 4 clearly demonstrates the impact of the pigment, where L* (lightness) values reveal a spectrum of color brightness. Fabrics with higher L* values appear lighter, while lower values indicate deeper shades. Impressively, polyamide achieves the highest L* value of 64.71, marking it as the lightest fabric when printed with this violet pigment. Following closely are cotton/polyester blends at 62.92 and cotton at 61.15, underscoring the pigment’s potential to enhance the visual appeal of various textiles. Wool, with L* value of 57.7, and polyester, at 59.79, demonstrate lower L* values, which results in richer, darker shades when printed. On the red-green axis, a* values reveal insightful shifts; positive values lean toward red, while negative values lean toward green. Notably, all fabrics exhibit positive a* values, highlighting how the violet pigment effectively enhances the color balance toward red. Among the various fabric types, the a* values are strikingly similar, with cotton (5.25) and polyamide (5.06) leading the way in vivid redness, making them standout choices for vibrant prints. b* (Yellow-Blue Axis), understanding color shifts can truly enhance our appreciation for different fabrics. Positive b* values signify a shift toward yellow, while negative values indicate a move toward blue. Notably, polyamide boasts the highest b* value at an impressive 11.95, demonstrating that the violet pigment on this fabric radiates a vibrant yellowish hue far more than any other fabric tested. Following closely, polyester and a cotton/polyester blend show b* values of 9.28 and 9.29, respectively, further reflecting these warmer tones. In contrast, cotton, with a b* value of 6.84, and wool, at 4.32, reveal a more neutral or blue-leaning tone, highlighting their color characteristics. When we look at *ΔE (Color Difference), which measures the perceptibility of color change from the original fabric to the printed version, cotton shines brightly. It demonstrates the most pronounced color change after being printed with the violet pigment, registering a ΔE* of 27.31. This striking difference emphasizes the fabric’s adaptability and appeal.

**Table 4:**
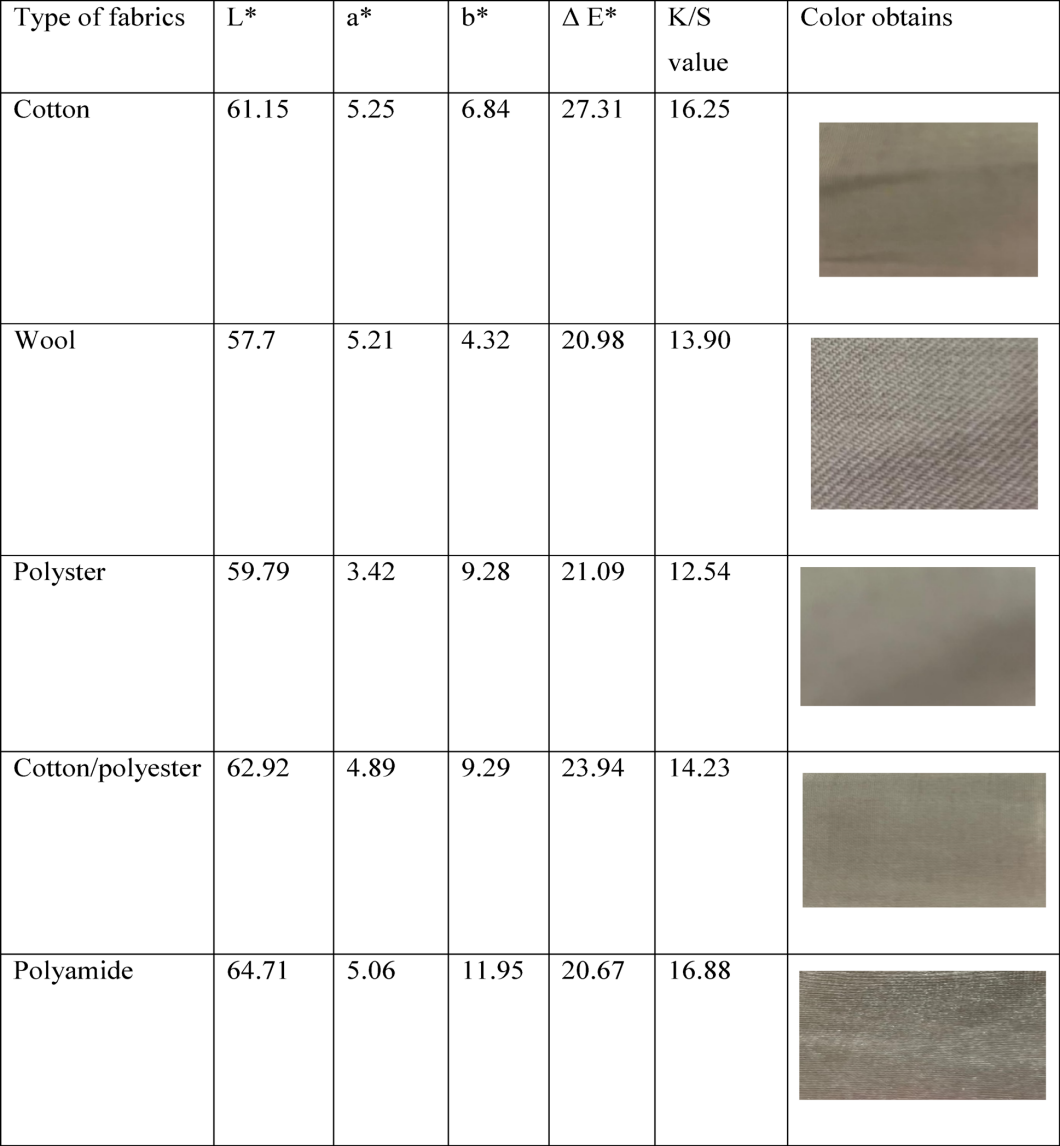
Color strengths and obtained colors of violet pigment extracted from *S. zaomycetitus* GH90 isolate on the fabrics

L*, a*, b* (the color coordinates), ΔE (color difference), and K/S value (color strength), L* (lightness), a* values (insightful shifts), b* (Yellow-Blue Axis), visible color change (ΔE*)

The K/S value, which indicates color strength and intensity, tells an equally compelling story. With a K/S value of 16.25, cotton stands out as the fabric that absorbs pigment most effectively, resulting in the richest color. Polyamide is not far behind at 16.88, showcasing its strong color intensity. Meanwhile, wool (13.90) and polyester (12.54) reflect lower K/S values, which suggest a subtler color application. The cotton/polyester blend strikes a balance with a K/S value of 14.23, lending it a moderate yet appealing color intensity that outperforms both polyester and wool. Together, these insights underscore the unique qualities of each fabric, making them worthy of your consideration. Cotton stands out with the highest color strength (K/S = 16.25), showcasing its exceptional ability to hold the violet pigment and deliver a strikingly vibrant hue. Polyamide also displays impressive color intensity (K/S = 16.88), attributed to its strong affinity for the pigment. However, its higher lightness (L*) results in a visibly lighter color. In comparison, polyester and wool exhibit lower K/S values, indicating reduced pigment absorption and, consequently, less intense colors. The ΔE values signify that cotton undergoes the most significant color transformation when printed with the violet pigment, while polyamide and wool reveal more subtle changes. Polyamide presents a lighter color due to its distinctive fiber characteristics, yet it also displays the most noticeable yellowish tone (b* = 11.95), subtly warming the violet hue. When considering responsiveness to the violet pigment, cotton and polyamide lead the way in both color intensity (K/S) and visible color change (ΔE*). The optimal fabric choice will hinge on achieving the right balance between striking color strength and aesthetic appeal across different materials. Polyester and wool’s more muted effects may restrict their use in applications demanding vibrant color intensity. The violet pigment derived from the *S. zaomycetitus* strain GH90 represents a game-changing opportunity for textile dyeing, especially with cotton and polyamide at the forefront for color strength and intensity. The pigment’s remarkable ability to produce a noticeable change (ΔE*) and its high K/S values—particularly on cotton—highlight its immense potential for textile industries focused on creating vivid and UV-protective fabrics that captivate consumers and elevate products [40, 74].

#### Fastness properties

Table 5 presented an investigation of the fastness properties of a violet pigment produced by *S. zaomycetitus* strain GH90. In this study, we are focusing on five types of fabrics: cotton, wool, polyester, polyamide, and a cotton/polyester blend. The fastness properties tested include wash fastness, perspiration fastness (acidic, alkaline, and acidic perspiration), and light fastness. The violet pigment produced by *S. zaomycetitus* strain GH90 demonstrates strong fastness properties across multiple fabric types, making it a promising candidate for textile applications. The pigment performs well in wash fastness, with 3–4 or better results for all fabrics, ensuring durability through repeated washing. Perspiration fastness: particularly in alkaline conditions, the pigment shows excellent resistance to fading, with 4–5 results across all fabrics. Lightfastness of pigment excels in lightfastness, showing 5–6 on most fabrics and 6–7 on polyamide, indicating good resistance to sunlight fading. The polyamide fabric shows the best overall performance in terms of color intensity (K/S) and light fastness, followed by cotton. The cotton/polyester blend fabric, while slightly lower in color intensity, also performs well in fastness tests, suggesting that the violet pigment can be effectively applied to a variety of textile materials with good durability. Thus, *S. zaomycetitus* strain GH90’s violet pigment is a viable option for producing vibrant and durable prints on textiles, particularly in applications requiring good fastness properties [75].

**Table 5:** Investigation of fastness properties of a Violet pigment produced by *S. zaomycetitus* strain GH90

Type of fabrics			Cotton	Wool	Polyester	Cotton/polyester blend	Polyamide
K/SValues			16.25	13.90	12.54	14.23	16.88
Wash fastness		Alt.	3–4	4	3–4	3–4	3–4
		St.	4	4	4	3–4	3–4
Perspiration fastness	Acide	Alt.	3	3	3–4	3	3–4
		St.	3	3–4	3	3–4	3–4
	Alkali	Alt.	4–5	5	5	4–5	4–5
		St.	4–5	4–5	4–5	4–5	4–5
Light fastness			5–6	6	5–6	6	6–7

K/S value (color intensity)

#### UV protection assessment

The study you have outlined focuses on the isolation and molecular identification of *S. zaomycetitus* strain GH90, a novel violet pigment producer, and its potential applications in enhancing the Ultraviolet Protection Factor (UPF) of various textile fibers. The UPF values were measured at different concentrations of the violet pigment (GH90) on a range of fabrics, including wool, cotton, polyester, polyamide, and a cotton/polyester blend as shown in Table 6.

**Table 6:** The effect of different concentration of violet pigment GH90 on the UPF of natural, synthetic and blend fabrics

Type of sample	UPF at different concentration of violet pigment produced by S. zaomycetitus GH90.			
Concentration of pigment GH8	0.5%	1%	1.5%	2%
Wool	55.43	65.76	73.65	72.34
Cotton	23.87	33.63	49.5	54.6
Polyester	61.64	61.99	72.58	74.34
Cotton/polyester	62.32	53.24	62.93	63.98
Polyamide	65.34	64.43	77.37	78.19

*UPF* The Ultraviolet Protection Factor

From Table 5, the concentration of the violet pigment increased (0.5–2%), and there was a general increase in the UPF across all fabric types. The highest increase in UPF was observed with polyamide and polyester fabrics at 2% concentration, indicating that these fibers were most responsive to the pigment’s UV-blocking properties. The UPF of wool fabrics increased steadily from 55.43 at 0.5% to a peak of 73.65 at 1.5%, but slightly decreased to 72.34 at 2%. This suggests that the optimal concentration for wool might be around 1.5%, but in the cotton, UPF increased from 23.87 at 0.5% to 54.6 at 2%, with the most significant improvement between 1% and 2% concentration. Polyester showed a moderate increase in UPF, from 61.64 at 0.5% to 74.34 at 2%. The highest UPF was recorded at 2%. The UPF of the blend fabric increased from 62.32 at 0.5% to 63.98 at 2%, showing a relatively stable enhancement with pigment concentration. Polyamide fabric exhibited the most significant increase, starting at 65.34 at 0.5% and peaking at 78.19 at 2%. This fabric showed the greatest overall improvement in UPF across all concentrations. *S. zaomycetitus* strain GH90’s violet pigment is a promising natural alternative for enhancing the UV protection of textiles, particularly for fabrics like polyamide and polyester, where it provides significant protection against harmful UV radiation [21].

### Antibacterial properties of treated fabrics

**Fig. 7:**
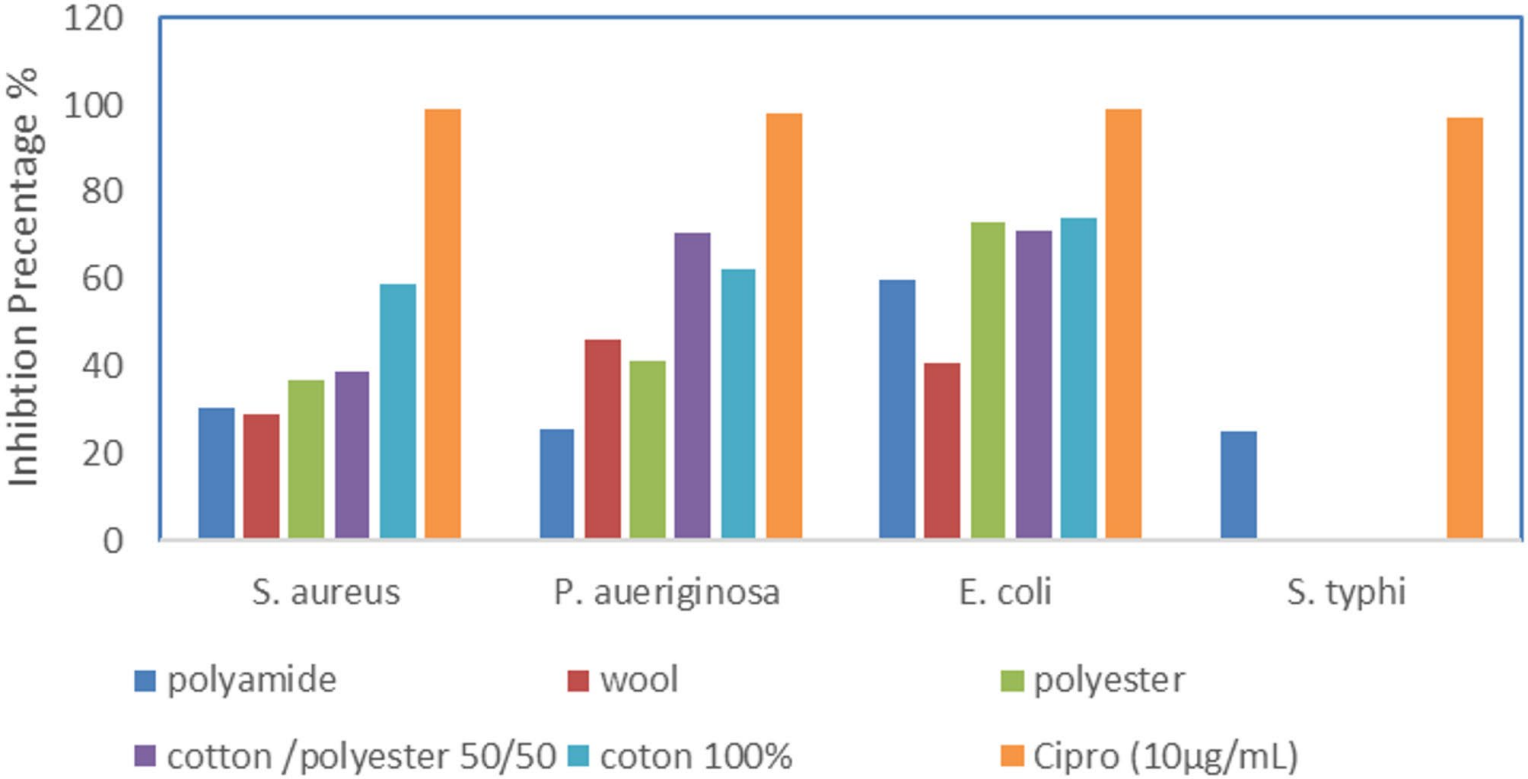
Antibacterial activity of treated fabrics

The results indicate a differential inhibition of bacterial strains based on the type of textile treatment. *Staphylococcus aureus* and *Pseudomonas aeruginosa* showed the highest inhibition in 100% cotton (58.60% and 62.24%, respectively), while *Escherichia coli* exhibited strong inhibition across polyester, cotton/polyester, and 100% cotton, with 100% cotton showing the highest at 73.81%. *Salmonella typhi* was not inhibited by most textile treatments, except for polyamide (25.22%), indicating selective activity. The control, ciprofloxacin (10 µg/mL), showed the highest inhibition for all strains, with *S. aureus* reaching 99.25%. The effectiveness of textiles against different bacterial species varies significantly, with 100% cotton showing broader antimicrobial efficacy (Fig. 7).

### ADME-physiochemical properties of most abundant compound

**Fig. 8:**
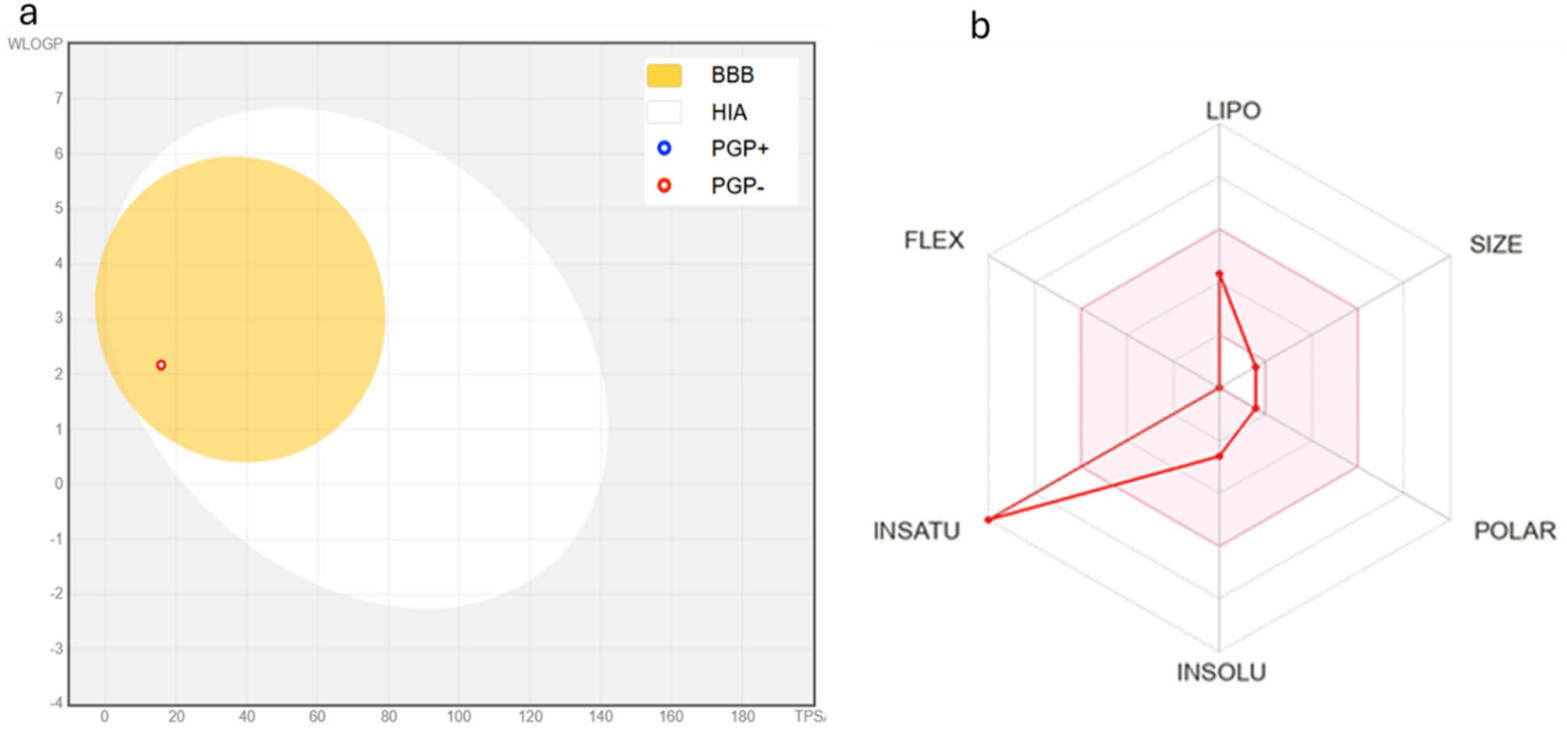
**a** Boiled egg chart **b** Bioavailability radar chart

The most abundant compound among the mentioned ones is indole, which is often found as a major metabolite in various biological systems, especially in bacteria, plants, and fungi. It serves as a precursor for many sondary metabolites. Indole, a compound with the molecular formula C₈H₇N and a molecular weight of 117.15 g/mol, exhibits unique physicochemical properties that influence its pharmacokinetic profile. Structurally, it consists of nine heavy atoms, all of which are aromatic, and has a complete absence of aliphatic carbons as indicated by a Fraction Csp³ of 0.00. This feature contributes to its rigid, planar aromatic structure, which plays a key role in interactions with biological targets. The compound lacks rotatable bonds, which suggests limited conformational flexibility, potentially enhancing its binding specificity but reducing entropy-driven adaptability. The hydrogen bonding profile of indole is also distinctive. It contains a single hydrogen bond donor and no hydrogen bond acceptors, with a total polar surface area (TPSA) of 15.79 Å². Such a low TPSA ensures that indole remains highly permeable across biological membranes, aligning with its predicted high gastrointestinal absorption and ability to cross the blood-brain barrier (BBB). This property is critical for compounds targeting central nervous system (CNS) disorders or requiring systemic bioavailability (Fig. 8a, b).

Indole demonstrates moderate lipophilicity across different calculation models, with a Consensus Log P (partition coefficient) of 1.98. Individual Log P values range from 1.43 (iLOGP) to 2.66 (SILICOS-IT), suggesting that it has sufficient lipophilicity to favor membrane permeability while avoiding excessive accumulation in lipid-rich environments. This balanced lipophilicity is advantageous in maintaining solubility and bioavailability. The solubility of indole is classified as soluble, with values ranging from 0.07 mg/ml (SILICOS-IT) to 1.14 mg/ml (Ali model). Such solubility levels support its formulation potential for various drug delivery systems. From a pharmacokinetics perspective, indole is not a substrate for P-glycoprotein (P-gp), implying minimal efflux from cells, which enhances its bioavailability. Interestingly, it is predicted to inhibit CYP1A2, one of the key drug-metabolizing enzymes in the liver, while showing no inhibitory effects on other cytochrome P450 enzymes like CYP2C19, CYP2C9, CYP2D6, and CYP3A4. This selective inhibition may influence the metabolism of co-administered drugs, highlighting its potential role in drug-drug interactions.

Indole satisfies most drug-likeness criteria, with Lipinski’s Rule of Five showing no violations. However, it fails to meet the criteria set by Ghose and Muegge rules due to its low molecular weight and limited structural complexity. Its bioavailability score of 0.55 suggests moderate oral bioavailability, which is supported by its high gastrointestinal absorption. The synthetic accessibility score of 1.00 further highlights its ease of chemical synthesis, making it an attractive lead compound for medicinal chemistry optimization.

In terms of toxicity, indole shows relatively low potential for severe adverse effects. It has minimal predicted hERG blocking liability (0.12) and negligible likelihood of clinical toxicity (0.01). However, its mutagenicity score (0.34) and drug-induced liver injury potential (0.78) warrant consideration during preclinical safety evaluations. Indole’s propensity to interact with the aryl hydrocarbon receptor (0.71) may be relevant for its effects on xenobiotic metabolism and environmental toxicity. Overall, the physicochemical properties and ADME profile of indole suggest a compound with excellent permeability, moderate solubility, and manageable toxicity risks. These characteristics make it a promising candidate for drug development, particularly for CNS-active or gastrointestinal-targeted therapies. However, its enzyme inhibition profile and potential toxicological interactions require careful evaluation during lead optimization and preclinical studies.

### Molecular docking simulation between the most abundant compound and OMPA and OprD

**Fig. 9:**
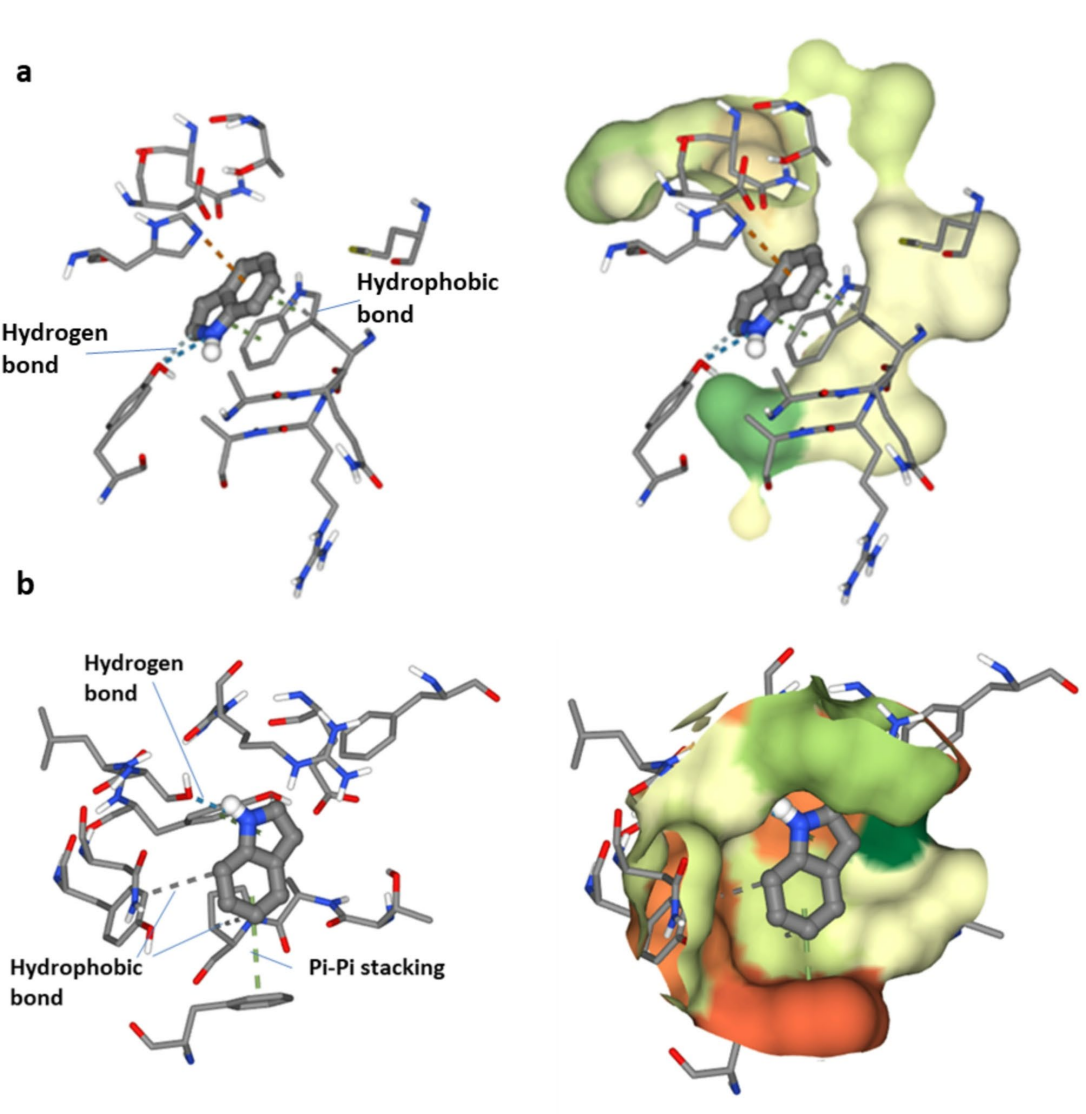
Molecular docking simulation between the most abundant compound and **a** *E. coli* OMPA and **b** *P. aeruginosa* OprD

The docking simulation of indole with Outer Membrane Protein A (OMPA) (PDB ID: 1BXW) has a binding affinity of −6.4 kcal/mol, indicating a robust contact between the ligand and the receptor. Hydrophobic interactions between the ligand atom C7 and Tryptophan 102 (CB) indicate a stable nonpolar contact. Moreover, π-π stacking interactions between the ligand’s C7 and C2 atoms and the indole ring of Tryptophan 102 (NE1 and CZ3) augment the binding affinity via aromatic interactions. A cation-π interaction between the ligand’s C7 and histidine 19 (NE2) enhances the complex via electrostatic forces. The nitrogen atom (N1) of indole and the hydroxyl group of Tyrosine 72 (OH) form strong hydrogen bonds, strengthening binding. C6 of the ligand and Tyrosine 72′s hydroxyl group form a reduced hydrogen bond, stabilising it further. The binding affinity of −6.4 kcal/mol, along with these interactions, indicates that the indole-OMPA complex is thermodynamically favorable, with Tryptophan 102, Histidine 19, and Tyrosine 72 significantly contributing to binding stabilization through hydrophobic, electrostatic, and hydrogen bonding interactions. Pseudomonas aeruginosa OprD is crucial for the selective absorption of vital nutrients and the expulsion of antibiotics in Pseudomonas aeruginosa, making it a key target in combating bacterial resistance. This docking research reveals that indole may serve as a ligand that modulates OprD activity, offering a promising foundation for developing new treatment techniques to address antibiotic resistance in this pathogen. The molecular docking of indole with the crystal structure of Pseudomonas aeruginosa OprD demonstrated numerous notable interactions with critical amino acid residues. Hydrophobic interactions were noted between carbon atom C8 and proline (Pro123) and between carbon atom C4 and tyrosine (Tyr214). Pi-pi stacking interactions were observed between carbon atom C2 and tyrosine (Tyr202) and between carbon atom C7 and phenylalanine (Phe298). Additionally, robust hydrogen connections were established between nitrogen atom N1 and serine (Ser200), but a moderate hydrogen link was detected between carbon atom C6 and tyrosine (Tyr202). The binding energy of this interaction was determined to be −5.2 kcal/mol (Fig. 9a, b).

## Conclusion

In summary, the violet pigment produced by *S. zaomyceticus* GH90 presents significant industrial and medical applications. Optimizing production parameters enhanced pigment yield and stability across different temperatures and pH levels. The pigment’s antibacterial properties render it suitable for antimicrobial textile applications, while its stability and retention at elevated temperatures indicate its resilience. The primary pigment component may possess medicinal properties, as indicated by molecular research and docking studies, which highlight its permeability, solubility, and interactions with enzymes. The violet pigment produced by *S. zaomyceticus *GH90 serves as a sustainable alternative to synthetic dyes and exhibits potential applications in textiles and medicine. Further research is necessary to explore its extensive biotechnological applications.

## Supplementary Information

Below is the link to the electronic supplementary material.

Supplementary Material 1

## Data Availability

The Streptomyces isolate identified in this study was submitted to the NCBI Gene Bank database under accession number OQ146749.1. The link (https://www.ncbi.nlm.nih.gov/search/all/?term=OQ146749.1).
