# Supplementary material for: Streptomyces zaomycetitus strain GH90: a source of violet pigment with metabolic profiling and potential application in textile: in vitro supported by in silico studies and molecular docking
Source: BMC Microbiol. 2025 Oct 3;25:627. doi: 10.1186/s12866-025-04329-1 (PMC12492711; doi:10.1186/s12866-025-04329-1)
Supplement: Supplementary file 1 — Supplementary Material 1 [file 12866_2025_4329_MOESM1_ESM.docx]

Supplementary Table 1. ADME physicochemical properties of Indole.

| **Property** | **Value** |
| --- | --- |
| **Molecular Formula** | C₈H₇N |
| **Molecular Weight** | 117.15 g/mol |
| **Number of Heavy Atoms** | 9 |
| **Number of Aromatic Heavy Atoms** | 9 |
| **Fraction Csp³** | 0.00 |
| **Number of Rotatable Bonds** | 0 |
| **Number of H-bond Acceptors** | 0 |
| **Number of H-bond Donors** | 1 |
| **Molar Refractivity** | 38.30 |
| **Polar Surface Area (TPSA)** | 15.79 Å² |
| **Log Po/w (iLOGP)** | 1.43 |
| **Log Po/w (XLOGP3)** | 2.05 |
| **Log Po/w (WLOGP)** | 2.17 |
| **Log Po/w (MLOGP)** | 1.57 |
| **Log Po/w (SILICOS-IT)** | 2.66 |
| **Consensus Log Po/w** | 1.98 |
| **Log S (ESOL)** | -2.60 |
| **Solubility (ESOL)** | 2.96e-01 mg/ml; 2.52e-03 mol/l |
| **Log S (Ali)** | -2.01 |
| **Solubility (Ali)** | 1.14e+00 mg/ml; 9.77e-03 mol/l |
| **Log S (SILICOS-IT)** | -3.23 |
| **Solubility (SILICOS-IT)** | 6.90e-02 mg/ml; 5.89e-04 mol/l |
| **GI Absorption** | High |
| **BBB Permeant** | Yes |
| **P-gp Substrate** | No |
| **CYP1A2 Inhibitor** | Yes |
| **CYP2C19 Inhibitor** | No |
| **CYP2C9 Inhibitor** | No |
| **CYP2D6 Inhibitor** | No |
| **CYP3A4 Inhibitor** | No |
| **Log Kp (Skin Permeation)** | -5.56 cm/s |
| **Lipinski Rule Violation** | No violation (meets Lipinski’s Rule of Five) |
| **Ghose Rule Violation** | 3 violations (MW<160, MR<40, #atoms<20) |
| **Veber Rule** | Yes |
| **Egan Rule** | Yes |
| **Muegge Rule Violation** | 2 violations (MW<200, Heteroatoms<2) |
| **Bioavailability Score** | 0.55 |
| **PAINS Alert** | 0 alert |
| **Brenk Alert** | 0 alert |
| **Leadlikeness Violation** | 1 violation (MW<250) |
| **Synthetic Accessibility** | 1.00 |
| **hERG Blocking** | 0.12 (32.38% percentile) |
| **Clinical Toxicity** | 0.01 (10.00% percentile) |
| **Mutagenicity** | 0.34 (69.91% percentile) |
| **Drug-Induced Liver Injury** | 0.78 (69.79% percentile) |
| **Carcinogenicity** | 0.03 (11.13% percentile) |
| **Acute Toxicity LD50** | 2.24 (32.42% percentile) log(1/(mol/kg)) |
| **Skin Reaction** | 0.73 (78.60% percentile) |
| **Androgen Receptor (Full Length)** | 0.01 (19.58% percentile) |
| **Androgen Receptor (Ligand Binding Domain)** | 3.04e-03 (22.99% percentile) |
| **Aryl Hydrocarbon Receptor** | 0.71 (96.74% percentile) |
| **Aromatase** | 0.02 (48.51% percentile) |
| **Estrogen Receptor (Full Length)** | 0.10 (54.79% percentile) |
| **Estrogen Receptor (Ligand Binding Domain)** | 0.02 (48.89% percentile) |
| **Peroxisome Proliferator-Activated Receptor Gamma** | 3.19e-03 (42.85% percentile) |
| **Nuclear Factor (Erythroid-Derived 2)-Like 2/Antioxidant Responsive Element** | 0.05 (29.43% percentile) |
| **ATPase Family AAA Domain-Containing Protein 5 (ATAD5)** | 0.02 (68.71% percentile) |
| **Heat Shock Factor Response Element** | 0.09 (84.10% percentile) |
| **Mitochondrial Membrane Potential** | 0.05 (54.98% percentile) |
| **Tumor Protein p53** | 0.01 (41.84% percentile) |


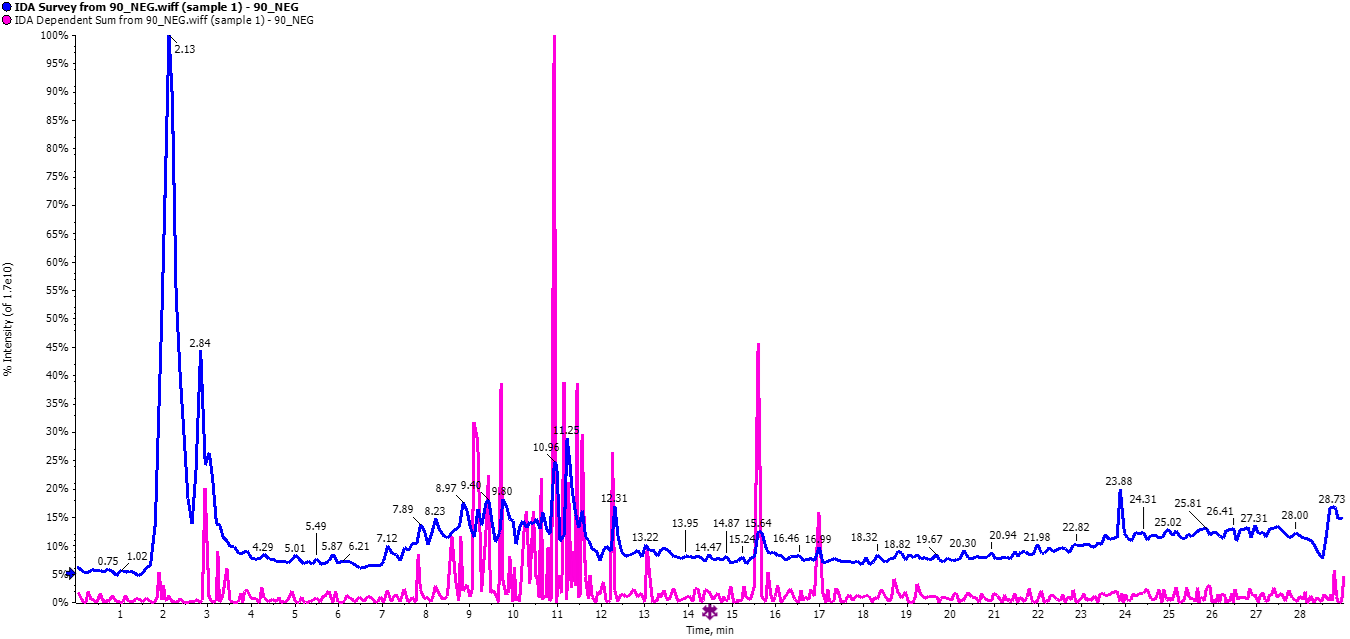


Figure. LCMASS chromatogram of the pigment (NEGATIVE MODE)

Table. LCMASS profile of the pigment (NEGATIVE MODE)

| Negative mode |  |  |  |  |  |  |  |  |  |
| --- | --- | --- | --- | --- | --- | --- | --- | --- | --- |
| ID | RT (min) | m/z | Type | Metabolite name |  | Height | Area | Gaussian | S/N |
| 102 | 0.912867 | 165.9918 | [M-H]- | w/o MS2:Pyridoxal |  | 1616506 | 3.74E+07 | 0.789697 | 3540.35 |
| 125 | 1.024183 | 195.915 | [M-H]- | w/o MS2:3,4-Dihydroxy-L-phenylalanine |  | 1573415 | 4.60E+07 | 0.772751 | 5.606815 |
| 195 | 1.410767 | 149.0844 | [M-H]- | w/o MS2:D-(-)-Arabinose |  | 1188321 | 4.13E+07 | 0.796072 | 1.063956 |
| 1673 | 2.0281 | 102.8849 | [M-H]- | HydroxyButyric acid |  | 4626801 | 7.64E+07 | 0.938955 | 47.52283 |
| 1761 | 2.0281 | 277.1554 | [M+Na-2H]- | w/o MS2:gamma-Linolenic acid |  | 4819466 | 9.03E+07 | 0.59904 | 17.58802 |
| 2209 | 2.1144 | 130.8896 | [M-H2O-H]- | w/o MS2:Methylsuccinic acid |  | 5002325 | 6.83E+07 | 0.815776 | 99998.1 |
| 3895 | 2.372233 | 243.0675 | [M+Na-2H]- | w/o MS2:Uridine |  | 6318966 | 1.50E+08 | 0.874124 | 28.56546 |
| 3896 | 2.372233 | 243.1135 | [M+Na-2H]- | w/o MS2:Uridine |  | 6.32E+06 | 1.19E+08 | 0.576182 | 34.69929 |
| 4171 | 2.4537 | 175.2229 | [M-H]- | w/o MS2:Canavanine |  | 1.66E+07 | 4.74E+08 | 0.797279 | 58.24227 |
| 4377 | 2.47735 | 128.9741 | [M-H]- | w/o MS2:CITRACONIC ACID |  | 2872.375 | 3.33E+04 | 0.978905 | 44.35735 |
| 4379 | 2.54825 | 117.2175 | [M-H]- | w/o MS2:3-Hydroxyisovaleric acid |  | 1.31E+07 | 3.30E+08 | 0.811105 | 49.50242 |
| 4532 | 2.732217 | 218.214 | [M-H]- | w/o MS2:trans-Zeatin |  | 3201405 | 8.46E+07 | 0.757537 | 20.26546 |
| 5437 | 2.944233 | 131.2005 | [M-H]- | w/o MS2:2-Hydroxy-4-methylpentanoate |  | 2.80E+07 | 4.55E+08 | 0.739541 | 184.6508 |
| 5599 | 3.035517 | 269.0658 | [M-H]- | w/o MS2:Apigenin |  | 2.62E+06 | 5.32E+07 | 0.86589 | 9.315032 |
| 5881 | 3.611 | 101.0755 | [M-H]- | 2-Oxobutyric acid |  | 3543091 | 1.15E+08 | 0.59064 | 6.843745 |
| 5936 | 3.804817 | 116.1382 | [M-H]- | w/o MS2:Norvaline |  | 6631655 | 1.15E+08 | 0.785006 | 3.117524 |
| 6022 | 4.205717 | 145.215 | [M-H]- | w/o MS2:2-Methylglutaric acid |  | 1844134 | 5.63E+07 | 0.769727 | 9.978289 |
| 6041 | 4.29335 | 435.0974 | [M-2H]2- | w/o MS2:Phlorizin |  | 2421678 | 4.26E+07 | 0.845254 | 23.78863 |
| 6177 | 5.01025 | 144.1302 | [M-H]- | w/o MS2:L-beta-Homoleucine |  | 9420828 | 1.54E+08 | 0.881155 | 65.64644 |
| 6200 | 5.120867 | 201.1863 | [M-H]- | w/o MS2:Sebacate |  | 3689259 | 6.30E+07 | 0.75712 | 26.46046 |
| 6202 | 5.120867 | 245.0708 | [M-H]- | w/o MS2:gamma,gamma-Dimethyallyl pyrophosphate ammonium salt | | 2637667 | 4.95E+07 | 0.767833 | 19.18859 |
| 6203 | 5.120867 | 245.1209 | [M-H]- | w/o MS2:gamma,gamma-Dimethyallyl pyrophosphate ammonium salt | | 2.64E+06 | 4.63E+07 | 0.857549 | 22.58533 |
| 6229 | 5.307734 | 219.9698 | [M-H]- | w/o MS2:N-Acetyl-D-mannosamine |  | 1105095 | 3.16E+07 | 0.845217 | 9910.87 |
| 6305 | 5.774333 | 178.1269 | [M-H]- | w/o MS2:L-beta-Homophenylalanine |  | 2.40E+06 | 5.48E+07 | 0.705859 | 10.28997 |
| 6312 | 5.774333 | 403.0821 | [M-2H]2- | w/o MS2:Uridine 5'-diphosphate |  | 1.65E+06 | 3.99E+07 | 0.742588 | 12.83049 |
| 6330 | 5.86735 | 147.0641 | [M-H]- | w/o MS2:Citramalic acid |  | 2.63E+06 | 7.05E+07 | 0.91454 | 1.511127 |
| 6343 | 5.86735 | 565.0915 | [M-2H]2- | w/o MS2:Uridine 5'-diphospho-D-glucose |  | 1.22E+06 | 2.37E+07 | 0.70169 | 14.69138 |
| 6368 | 6.091784 | 116.1261 | [M-H]- | w/o MS2:Norvaline |  | 3.95E+06 | 8.96E+07 | 0.989001 | 19.04738 |
| 6371 | 6.091784 | 165.9993 | [M-H]- | w/o MS2:Pyridoxal |  | 1574902 | 3.13E+07 | 0.920007 | 4399.731 |
| 6400 | 6.216367 | 148.8654 | [M-H]- | w/o MS2:L-(+)-Tartrate |  | 3684.125 | 5.55E+04 | 0.637553 | 59.62071 |
| 6414 | 6.370417 | 115.1141 | [M-H]- | Ketovaline |  | 3406592 | 8.86E+07 | 0.834648 | 3.745967 |
| 6685 | 7.502617 | 166.0036 | [M-H]- | w/o MS2:Pyridoxal |  | 1594890 | 5.13E+07 | 0.885761 | 9918.644 |


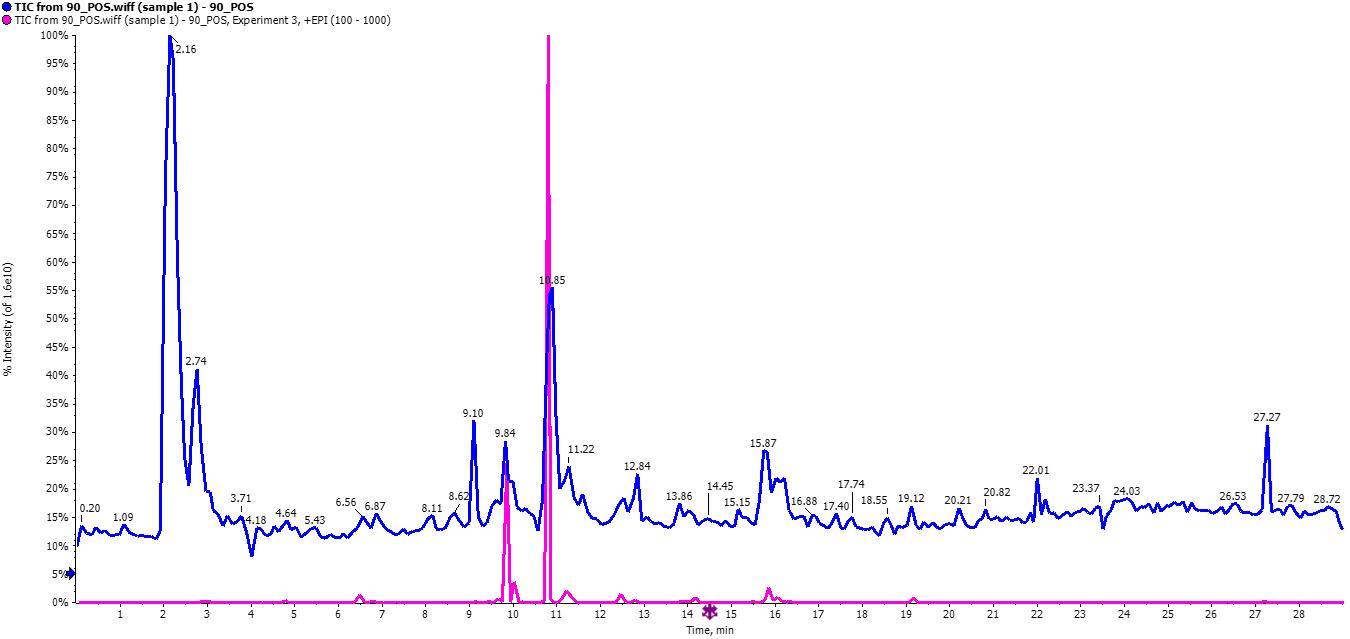


Figure. LCMASS chromatogram of the pigment (POSITIVE MODE)

Table. LCMASS profile of the pigment (POSITIVE MODE)

| Positive mode |  |  |  |  |  |  |  |  |  |
| --- | --- | --- | --- | --- | --- | --- | --- | --- | --- |
| ID | RT (min) | m/z | Type | Metabolite name |  | Height | Area | Gaussian | S/N |
| 2 | 0.357817 | 113.9791 | [M+H]+ | Creatinine |  | 3970304 | 1.11E+08 | 0.678839 | 1064.473 |
| 1018 | 2.138267 | 112.8772 | [M+H]+ | w/o MS2:URACIL |  | 1.89E+07 | 3.46E+08 | 0.832333 | 51.24752 |
| 1019 | 2.138267 | 112.9166 | [M+H]+ | w/o MS2:URACIL |  | 1.89E+07 | 3.26E+08 | 0.822423 | 59.40641 |
| 1024 | 2.138267 | 122.9313 | [M+H]+ | w/o MS2:BENZOIC ACID |  | 1.54E+07 | 3.17E+08 | 0.777876 | 20.46345 |
| 1028 | 2.138267 | 156.8657 | [M+Na]+ | w/o MS2:Orotic acid |  | 6435936 | 1.40E+08 | 0.83568 | 93295.88 |
| 1029 | 2.138267 | 156.9203 | [M+H]+ | w/o MS2:Orotic acid |  | 6435936 | 1.24E+08 | 0.79748 | 11.96014 |
| 1987 | 2.214483 | 140.0831 | [M+H]+ | w/o MS2:Tropinone |  | 1.01E+07 | 1.89E+08 | 0.837547 | 17.86525 |
| 1988 | 2.214483 | 140.2352 | [M+H]+ | w/o MS2:Tropinone |  | 1.01E+07 | 1.69E+08 | 0.821273 | 39259.01 |
| 1990 | 2.214483 | 155.9822 | [M+2H]2+ | w/o MS2:Indole-3-acetonitrile |  | 3.69E+07 | 6.11E+08 | 0.810094 | 70.61111 |
| 1991 | 2.214483 | 156.1812 | [M+H]+ | w/o MS2:His |  | 3.69E+07 | 5.89E+08 | 0.800887 | 738224.1 |
| 2489 | 2.3069 | 118.02 | [M+H]+ | INDOLE |  | 5.16E+07 | 1.14E+09 | 0.753571 | 57.24887 |
| 2500 | 2.3069 | 227.9299 | [M+H]+ | w/o MS2:2'-Deoxycytidine |  | 9315569 | 1.59E+08 | 0.884424 | 17.65411 |
| 2830 | 2.399117 | 136.1946 | [M+H]+ | w/o MS2:Adenine |  | 2951062 | 4.78E+07 | 0.907536 | 8429.335 |
| 2831 | 2.399117 | 160.0917 | [M+H]+ | w/o MS2:N-Isovaleroylglycine |  | 6160666 | 1.45E+08 | 0.901839 | 23.08624 |
| 2838 | 2.399117 | 181.0132 | [M+H]+ | w/o MS2:4-Hydroxyphenylpyruvic acid |  | 4378639 | 9.62E+07 | 0.750606 | 5.29417 |
| 2841 | 2.399117 | 190.0178 | [M+H]+ | w/o MS2:Quisqualate |  | 2.53E+07 | 4.90E+08 | 0.926908 | 95.11205 |
| 3023 | 2.571017 | 124.0263 | [M+H]+ | w/o MS2:NICOTINIC ACID |  | 1.71E+07 | 3.49E+08 | 0.822045 | 42.14206 |
| 3048 | 2.668483 | 130.0498 | [M+H]+ | w/o MS2:L-5-Oxoproline |  | 5762011 | 1.73E+08 | 0.906115 | 7.704378 |
| 3172 | 2.760967 | 113.0576 | [M+H]+ | URACIL |  | 7848230 | 1.59E+08 | 0.800043 | 21.06309 |
| 3174 | 2.760967 | 126.076 | [M+H]+ | 1-Methylhistamine |  | 7251119 | 1.48E+08 | 0.927024 | 15.63041 |
| 3176 | 2.760967 | 144.0398 | [M+H]+ | 4-METHYL-5-THIAZOLEETHANOL |  | 5439938 | 1.43E+08 | 0.836863 | 10.65519 |
| 3182 | 2.760967 | 162.0967 | [M+H]+ | w/o MS2:2-Aminoadipic acid |  | 5108378 | 1.16E+08 | 0.729693 | 7.745329 |
| 3198 | 2.760967 | 240.9716 | [M+H]+ | w/o MS2:L-Cystine |  | 2958869 | 5.64E+07 | 0.788512 | 6.707661 |
| 3552 | 2.8539 | 231.0403 | [M+H-H2O]+ | w/o MS2:D-Ribose 5-phosphate |  | 3876240 | 8.37E+07 | 0.934541 | 12.90226 |
| 3567 | 2.8539 | 324.9597 | [M+H]+ | w/o MS2:Uridine 5'-monophosphate |  | 3917893 | 7.26E+07 | 0.642062 | 11.16275 |
| 3643 | 2.963567 | 191.9572 | [M+H]+ | w/o MS2:5-Hydroxyindoleacetic acid |  | 2635990 | 4.93E+07 | 0.94546 | 8.943734 |
| 3653 | 2.963567 | 319.0272 | [M+H]+ | w/o MS2:Myricetin |  | 5021608 | 1.20E+08 | 0.905985 | 15.19568 |
| 3654 | 2.963567 | 319.1343 | [M+H]+ | w/o MS2:Zearalenone |  | 5021745 | 1.03E+08 | 0.655623 | 15.7522 |
| 3780 | 3.130533 | 166.9802 | [M+H]+ | w/o MS2:3-Methylxanthine |  | 2844491 | 5.98E+07 | 0.816554 | 41261.66 |
| 3784 | 3.130533 | 220.0219 | [M+H]+ | w/o MS2:4-Nitrophenyl phosphate |  | 1.03E+07 | 1.71E+08 | 0.799235 | 43.42266 |
| 3914 | 3.4545 | 187.0962 | [M+H]+ | w/o MS2:3-Phospho-D-glycerate |  | 5916980 | 1.17E+08 | 0.937877 | 13.46548 |
| 4023 | 3.665883 | 245.0069 | [M+H]+ | w/o MS2:alpha-L-(-)-Fucose 1-phosphate bis(cyclohexylammonium) salt | | 2876699 | 7.18E+07 | 0.789615 | 11.05636 |
| 4474 | 4.626033 | 174.9957 | [M+H]+ | w/o MS2:cis-Aconitate |  | 6233945 | 1.59E+08 | 0.710473 | 13.09991 |
| 4476 | 4.626033 | 192.9439 | [M+H]+ | w/o MS2:Scopoletin |  | 4542652 | 1.19E+08 | 0.657055 | 14.78759 |
| 4517 | 4.741066 | 146.0113 | [M+H]+ | w/o MS2:3-FORMYLINDOLE |  | 1.29E+07 | 2.57E+08 | 0.782969 | 50.81543 |
| 4907 | 5.485384 | 227.214 | [M+H]+ | w/o MS2:METHYL DIHYDROJASMONATE |  | 1594441 | 4.93E+07 | 0.914662 | 25222.16 |
| 5057 | 5.809683 | 170.9797 | [M+H-H2O]+ | w/o MS2:DL-Glyceraldehyde 3-phosphate |  | 1687652 | 3.59E+07 | 0.914716 | 2605.176 |
| 5112 | 5.922583 | 141.8683 | [M+H]+ | w/o MS2:Ethanolamine phosphate |  | 3864835 | 9.93E+07 | 0.879094 | 5152.443 |
| 5637 | 6.860816 | 147.104 | [M+2H]2+ | w/o MS2:L-(+)-Lysine |  | 2959345 | 1.48E+08 | 0.875752 | 30004.16 |
| 5654 | 6.860816 | 341.0425 | [M+H]+ | w/o MS2:alpha-D-Glucose-1,6-diphosphate |  | 2427212 | 4.96E+07 | 0.837416 | 5.047574 |
| 5702 | 6.9791 | 130.0994 | [M+H]+ | w/o MS2:Metformin |  | 3480944 | 1.04E+08 | 0.723831 | 3.016362 |
| 5717 | 6.9791 | 399.0553 | [M+H]+ | w/o MS2:S-Adenosyl-L-methionine |  | 3771142 | 9.30E+07 | 0.86246 | 5.623191 |
| 6267 | 8.040916 | 138.9727 | [M+H]+ | w/o MS2:P-HYDROXYBENZOIC ACID |  | 1855063 | 5.28E+07 | 0.740781 | 8749.084 |
| 6284 | 8.040916 | 388.0965 | [M+NH4]+ | w/o MS2:2'-Deoxycytidine-5'-diphosphate |  | 1.22E+07 | 2.32E+08 | 0.629364 | 45.16314 |
| 6285 | 8.040916 | 388.102 | [M+NH4]+ | w/o MS2:2'-Deoxycytidine-5'-diphosphate |  | 1.22E+07 | 2.32E+08 | 0.759658 | 41.14635 |
| 6926 | 8.9854 | 170.016 | [M+H]+ | w/o MS2:NOREPINEPHIRINE |  | 3844260 | 8.21E+07 | 0.887292 | 8.908769 |
| 7324 | 9.513483 | 373.1025 | [M+H]+ | w/o MS2:Eleutheroside B |  | 2646318 | 4.21E+07 | 0.976753 | 9.646272 |
| 7399 | 9.621017 | 174.9637 | [M+H-H2O]+ | w/o MS2:cis-Aconitate |  | 1.98E+07 | 3.35E+08 | 0.737028 | 29.76987 |
| 7403 | 9.621017 | 192.976 | [M+H]+ | w/o MS2:Scopoletin |  | 1.31E+07 | 2.41E+08 | 0.890792 | 25.41063 |
| 7476 | 9.714867 | 349.2096 | [M+H]+ | w/o MS2:Inosine-5'-monophosphate |  | 4230537 | 7.29E+07 | 0.935747 | 18.17868 |
| 7555 | 9.822617 | 227.0865 | [M+H]+ | w/o MS2:Carnosine |  | 8865320 | 1.77E+08 | 0.833144 | 16.63823 |
| 7780 | 9.990267 | 608.0868 | [M+NH4]+ | w/o MS2:Uridine-5'-diphospho-N-acetylgalactosamine disodium salt | | 6723671 | 1.03E+08 | 0.801087 | 44.37912 |
| 7928 | 10.18865 | 190.0113 | [M+H]+ | w/o MS2:Quisqualate |  | 4125949 | 9.16E+07 | 0.954665 | 14.39404 |
| 8962 | 10.97382 | 345.2216 | [M+H]+ | w/o MS2:Maltitol |  | 833727 | 9926086 | 0.819643 | 4.956913 |
| 9471 | 11.49767 | 301.1354 | [M+H]+ | w/o MS2:3 5 7-trihydroxy-4'-methoxyflavone |  | 2281359 | 5.01E+07 | 0.894838 | 5.31933 |
| 10485 | 12.63278 | 380.011 | [M+H]+ | w/o MS2:S-Lactoylglutathione |  | 3593992 | 8.46E+07 | 0.773286 | 18.73966 |
| 10600 | 12.75145 | 398.9771 | [M+H]+ | w/o MS2:S-Adenosyl-L-methionine |  | 5147027 | 1.24E+08 | 0.817714 | 17.03989 |
| 11496 | 13.90435 | 607.9331 | [M+2H]2+ | w/o MS2:Uridine-5'-diphospho-N-acetylgalactosamine disodium salt | | 4406363 | 7.70E+07 | 0.7864 | 40.9651 |
| 11563 | 14.0208 | 162.9977 | [M+H]+ | w/o MS2:3-Hydroxy-3-Methylglutaric acid |  | 7827918 | 1.65E+08 | 0.757352 | 16.773 |
| 11649 | 14.13525 | 141.9798 | [M+H]+ | Histidinol |  | 3615863 | 1.08E+08 | 0.884508 | 2.218822 |
| 11668 | 14.13525 | 446.9876 | [M+H]+ | w/o MS2:Baicalein-7-O-glucuronide |  | 7366970 | 1.15E+08 | 0.914531 | 21.29335 |
| 11792 | 14.34787 | 233.0261 | [M+H]+ | w/o MS2:Melatonin |  | 2136034 | 3.42E+07 | 0.908471 | 5.212296 |
| 12054 | 14.76597 | 260.1087 | [M+H]+ | D-Glucosamine-6-phosphate |  | 1656755 | 2.41E+07 | 0.800809 | 13.80174 |
| 12382 | 15.24235 | 228.0444 | [M+H]+ | w/o MS2:2'-Deoxycytidine |  | 1.05E+07 | 2.90E+08 | 0.838438 | 30.04419 |
| 12659 | 15.73657 | 142.2617 | [M+H]+ | w/o MS2:Histidinol |  | 1233272 | 1.40E+07 | 0.744443 | 11080.61 |
| 12708 | 15.73657 | 306.1529 | [M+H]+ | w/o MS2:Capsaicin |  | 4179088 | 7.38E+07 | 0.749933 | 23.35629 |
| 12709 | 15.73657 | 306.2048 | [M+H]+ | w/o MS2:Capsaicin |  | 4179088 | 7.36E+07 | 0.776743 | 33.58492 |
| 13111 | 16.2119 | 387.0768 | [M+H]+ | w/o MS2:1-O-b-D-glucopyranosyl sinapate |  | 5854542 | 9.60E+07 | 0.902972 | 23.85102 |
| 13194 | 16.31582 | 399.0125 | [M+H]+ | w/o MS2:S-Adenosyl-L-methionine |  | 6012565 | 1.11E+08 | 0.779062 | 8.33634 |
| 13290 | 16.53957 | 176.9521 | [M+H]+ | w/o MS2:N-Carbamoyl-L-Aspartic acid |  | 2927319 | 4.88E+07 | 0.958865 | 3.258924 |
| 14312 | 18.22138 | 136.0943 | [M+H]+ | w/o MS2:Adenine |  | 1521467 | 4.55E+07 | 0.699245 | 8341.878 |
| 15889 | 20.82293 | 148.9676 | [M+H]+ | w/o MS2:Citramalate |  | 2.60E+07 | 5.62E+08 | 0.825099 | 18.71281 |
| 16620 | 22.00025 | 403.0609 | [M+H]+ | w/o MS2:Thymidine-5'-diphosphate |  | 9948544 | 1.32E+08 | 0.92796 | 24.88681 |
| 17345 | 23.15298 | 128.0604 | [M+H]+ | w/o MS2:Isoguvacine |  | 1.53E+07 | 3.57E+08 | 0.873938 | 0.392654 |
| 18224 | 24.44592 | 161.1006 | [M+H]+ | w/o MS2:3-(2-AMINOETHYL)INDOLE |  | 2531685 | 4.58E+07 | 0.817449 | 890.2741 |
| 19368 | 26.21845 | 127.9546 | [M+H]+ | w/o MS2:Isoguvacine |  | 1.82E+07 | 4.05E+08 | 0.828375 | 13879.87 |
| 19729 | 26.66042 | 142.0802 | [M+H]+ | w/o MS2:Histidinol |  | 9461894 | 2.38E+08 | 0.666963 | 0.731053 |
| 20111 | 27.27025 | 161.1036 | [M+H]+ | w/o MS2:3-(2-AMINOETHYL)INDOLE |  | 3125690 | 7.16E+07 | 0.622288 | 2157.853 |
| 21112 | 28.38742 | 161.097 | [M+H]+ | w/o MS2:L-beta-homoglutamine-HCl |  | 3145902 | 1.18E+08 | 0.801411 | 2243.206 |
| 21423 | 28.73943 | 142.0799 | [M+H]+ | w/o MS2:Histidinol |  | 8112420 | 2.56E+08 | 0.716029 | 1.53724 |
